# Development of Suppressed Ion Chromatography for the Online Quantification of Cations in Electrochemical Ammonia Synthesis Research

**DOI:** 10.1002/cssc.202501974

**Published:** 2026-02-02

**Authors:** Sebastian Bragulla, Julian Lorenz, Corinna Harms, Michael Wark, K. Andreas Friedrich

**Affiliations:** ^1^ Electrochemical Energy Technology Institute of Engineering Thermodynamics Deutsches Zentrum für Luft‐ und Raumfahrt e. V. (DLR) German Aerospace Center Oldenburg Germany; ^2^ Institute for Building Energetics Thermotechnology and Energy Storage (IGTE) University of Stuttgart Stuttgart Germany; ^3^ Faculty V – Mathematics and Natural Sciences Institute of Chemistry Chemical Technology 1 Carl von Ossietzky University Oldenburg Germany; ^4^ Electrochemical Energy Technology Institute of Engineering Thermodynamics Deutsches Zentrum für Luft‐ und Raumfahrt e. V. (DLR) German Aerospace Center Stuttgart Germany

**Keywords:** analytical methods, electrochemistry, ion chromatography, nitrogen, renewable resources

## Abstract

Practical research on the electrochemical nitrogen reduction reaction (eNRR) requires quantitative ammonia trace analysis because production rates are on the order of µg h^−1^ in aqueous electrolyte. This challenge is further aggravated by complex sample matrices. Ion chromatography is a powerful analytical technique for the quantitative determination of ammonium down to ppb‐concentrations, but requires matrix elimination (ME) for these kinds of sample. We developed a suppressed cation chromatography method using automated matrix neutralization and ME to quantitatively determine ammonium in 0.2 M sulfuric acid electrolyte at µg L^−1^ concentrations for use in NRR research. Although direct conductivity detection of cations is less sensitive than unsuppressed indirect conductivity detection, baseline noise requires suppression at these concentrations. Nonlinearity of the calibration curve became noticeable below ≈ 1 ng ammonium. A method limit of detection of 2 µg L^−1^ (ppb_mol_) for ammonium was achieved at 100 µL injection volume. Direct coupling of the electrochemical cell and IC enabled online quantification. This online measurement of ammonium in 0.2 M sulfuric acid electrolyte revealed ammonium contamination rapidly liberated from the hitherto judged negligible Nafion ionomer of the gas diffusion electrode at open circuit voltage, showing prior production rates to be likely false positives.

## Graphical abstract

We developed a suppressed ion chromatography method with automated matrix neutralization and matrix elimination for the quantitative determination of ammonium in electrochemical ammonia synthesis research. Online determination of ammonium in 0.2 M sulfuric acid electrolyte revealed Nafion ionomer as a non‐negligible secondary source of ammonium contamination from airborne ammonia pollution.

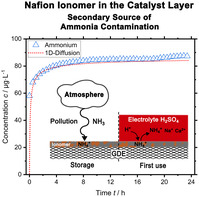

## 1 Introduction

Ammonia is an essential basic chemical indispensable for today's industry and modern agriculture. The industrial‐scale production of ammonia via the Haber–Bosch process with an estimated worldwide production of 150 million metric tons in 2024 [1] has a large CO_2_ footprint and is currently dependent on fossil fuels. An on‐demand, direct synthesis of green ammonia from water, nitrogen, and renewable electricity via an electrochemical nitrogen reduction reaction (eNRR) is a promising prospective alternative. In this light, the eNRR is currently of high research interest, especially the search for highly active and selective novel catalyst materials [2, 3]. To date, relevant production rates and faradaic efficiencies have only been proven by the lithium‐mediated eNRR in non‐aqueous electrolyte, which uses the spontaneous reaction of metallic lithium with nitrogen for chemical activation of nitrogen [4]. However, practical research on the eNRR for an electrochemical ammonia synthesis (EAS) in aqueous electrolyte hitherto necessitates quantitative trace analysis of ammonia (ammonium), because low ammonia production rates on the order of µg h^−1^ result to date in low analyte quantities within practical experimental duration. This challenge of small analyte concentrations is further aggravated by complex sample matrices, sample handling, and importantly, imperceptible contamination by ubiquitous environmental sources [5].

The majority of laboratories studying the eNRR use accessible spectrometric techniques with a color producing reaction (Nessler's reagent, indophenol blue) or ^1^H nuclear magnetic resonance (NMR) spectroscopy for the quantitative detection of ammonia [6]. However, these spectrometric techniques are sensitive to environmental factors such as sample pH and cationic interference [7]. ^1^H‐NMR enables isotope‐sensitive detection, but is complex and requires expensive equipment for sufficient detection [6]. Ion chromatography (IC) is a versatile and powerful analytical technique for the automated quantitative determination of ions such as ammonium. In a predecessor study, we showed that indirect conductivity detection of ammonium by unsuppressed IC enables fast and reliable quantification of ammonium down to 6 µg L^−1^ at an injection volume of 100 µL [8]. However, the highly acidic 0.1 M sulfuric acid sample matrix of these real samples required manual neutralization and a tenfold sample dilution. This dilution resulted in a proportionally higher effective quantification limit. It was not expedient to improve the limit of quantification further to counteract this due to an already marginal signal‐to‐noise ratio (SNR) and a maximized direct injection volume of 100 µL. The best avenue for further improvements is an automated matrix elimination (ME).

ME works by the selective retention of the analyte cations on a dedicated enrichment column, consisting of a weak acid cation (WAC) exchange resin, while the matrix is discarded. A prior matrix neutralization (MN) is required in case of highly acidic sample matrices, such as 0.1 M sulfuric acid, because WAC exchange resins such as the enrichment column are ionized only at a pH above 5–6. MN is implemented by a weak base anion (WBA) exchange resin, which neutralizes the acid and retains the acid anion. The strong acid eluent can be neutralized likewise after passing the separation column to chemically suppress it, which greatly improves the SNR, even though it does not improve the intrinsic detection sensitivity in case of cations. This direct conductivity detection of analyte cations enables reliable quantification below 1 µg L^−1^ (100 µL injection volume, 0.1 ng analyte). While a powerful analytical method is necessary for eNRR research, applying the method for online analysis will greatly improve the use and depth of generated results.

In this work, we develop a chemically suppressed cation chromatography method to quantitatively analyze ammonium in a 0.2 M sulfuric acid electrolyte sample matrix using automated MN and ME. In doing so, we explore the chromatographic system, the setup, and crucially, generate an analytical calibration curve to achieve a reliable quantification of ammonium at µg L^−1^ concentrations. This developed fast method is applied to real‐world eNRR experiments to quantify ammonium in sulfuric acid electrolyte to enhance the temporal resolution of eNRR data. Thus, ammonium production trajectories, whether by genuine production or contamination, are obtained during quantitative experiments, improving the understanding of catalytic processes. Direct coupling of the electrochemical cell setup and the ion chromatograph enables online quantification of ammonium with a time resolution of 30 min, giving new insights into the time dependency of the reaction and possible sources of contamination. In a first step, this online measurement of ammonium in 0.2 M sulfuric acid electrolyte by IC is applied to the quantification of background contamination of the used measurement setup at open circuit potential. This setup includes a gas diffusion electrode (GDE) sample coated with zirconium nitride nanoparticles, revealing the underestimated contribution of the Nafion ionomer of the catalyst layer to contamination.

## 2 Experimental

### 2.1 Chemicals and Labware

All water used was ultrapure water (Merck Milipore, Milli‐Q, >18.2 MΩ cm, 0.45 µm filter). All used chemicals were of reagent grade suitable for IC or higher if not specified otherwise: nitric acid, ROTIPURANSupra 69%, Carl Roth, Art. No. HN50; rubidium nitrate, 99.7% trace metal basis, MERCK, 289299; potassium carbonate, EMSURE, 1049280500; potassium hydrogencarbonate, Merck, EMSURE, 1048540500). All used cation standards, single element standards nominal concentration 1000 mg L^−1^ (MERCK, lithium, 59878; sodium, 43492; ammonium, 59755; potassium, 53337; magnesium, 89441; calcium, 39865) and multication standard 1 (MERCK, 91286), were of TraceCERT certified reference material grade. Elemental standards were stored refrigerated after opening.

### 2.2 Chromatographic System

The chromatographic system consisted of a Metrohm 850 Professional IC AnCat ion chromatograph (2.850.3030) with 942 Extension Modul Vario Prep 2 (2.942.0020) and 863 Compact IC Autosampler (2.863.0010). A Metrosep C 6 250/4.0 separation column (6.1051.430) with corresponding Metrosep C 6 Guard/4.0 (6.1051.500) guard column was used for separation. The separation column was additionally protected by a Metrosep RP 2 Guard/3.5 (6.1011.030) filter column. A Metrohm Suppressor Modul (MSM) HC Rotor C (6.2842.200) suppressor rotor for the MSM was used for chemical suppression. CO_2_ was sequentially suppressed by use of the Metrohm CO_2_ Suppressor. MN was achieved using the same suppressor concurrently, albeit a different chamber. A Metrosep C PCC 1 HC/4.0 enrichment column was used for ME. The extension module was equipped with a 100 µL PEEK sample loop. A Metrosep C Trap 1 (6.1015.000) trap column for cationic impurities purified the transfer solution, for which the detector effluent was used. The used Metrohm 863 Compact IC Autosampler (2.863.0010) is equipped with a peristaltic pump and peek needle for sample aspiration. The autosampler was connected to the IC using a 0.75 mm inner diameter PEEK capillary as recommended by Metrohm for trace analysis. Online measurements were realized by directly placing the inlet and outlet of PTFE tubing in the connected vessel in place of the autosampler. The respective liquid was constantly circulated through the sampling loop by a peristaltic pump at roughly 1 mL min^−1^.

### 2.3 Chromatographic Conditions

The eluent consisted of 5 mM nitric acid with 50 µg L^−1^ Rb^+^ ion stored in a 2 L polypropylene container sparged with helium. The separation column was kept at 40°C with the recommended flow rate of 0.9 mlmin^−1^. The resulting system pressure was 10.1 ± 0.1 MPa in normal operation fully equilibrated. The suppressor was regenerated with 70 mM potassium carbonate and 70 mM potassium bicarbonate and rinsed using detector effluent. Potassium salts were chosen, because potassium elutes after ammonium in contrast to sodium, which elutes directly before ammonium. The capacity per WBA exchange resin chamber is given by the manufacturer as 0.25 meq, which is sufficient for 55 min of eluent suppression at these conditions. The suppressor chambers were rotated every 20 min, except during chromatograms, a full rotation took 60 min. The chromatogram duration was 25 min to allow for complete elution of Ca^2+^ traces at these chromatographic conditions.

## 3 Results and Discussion

### 3.1 Method Development

**FIGURE 1:**
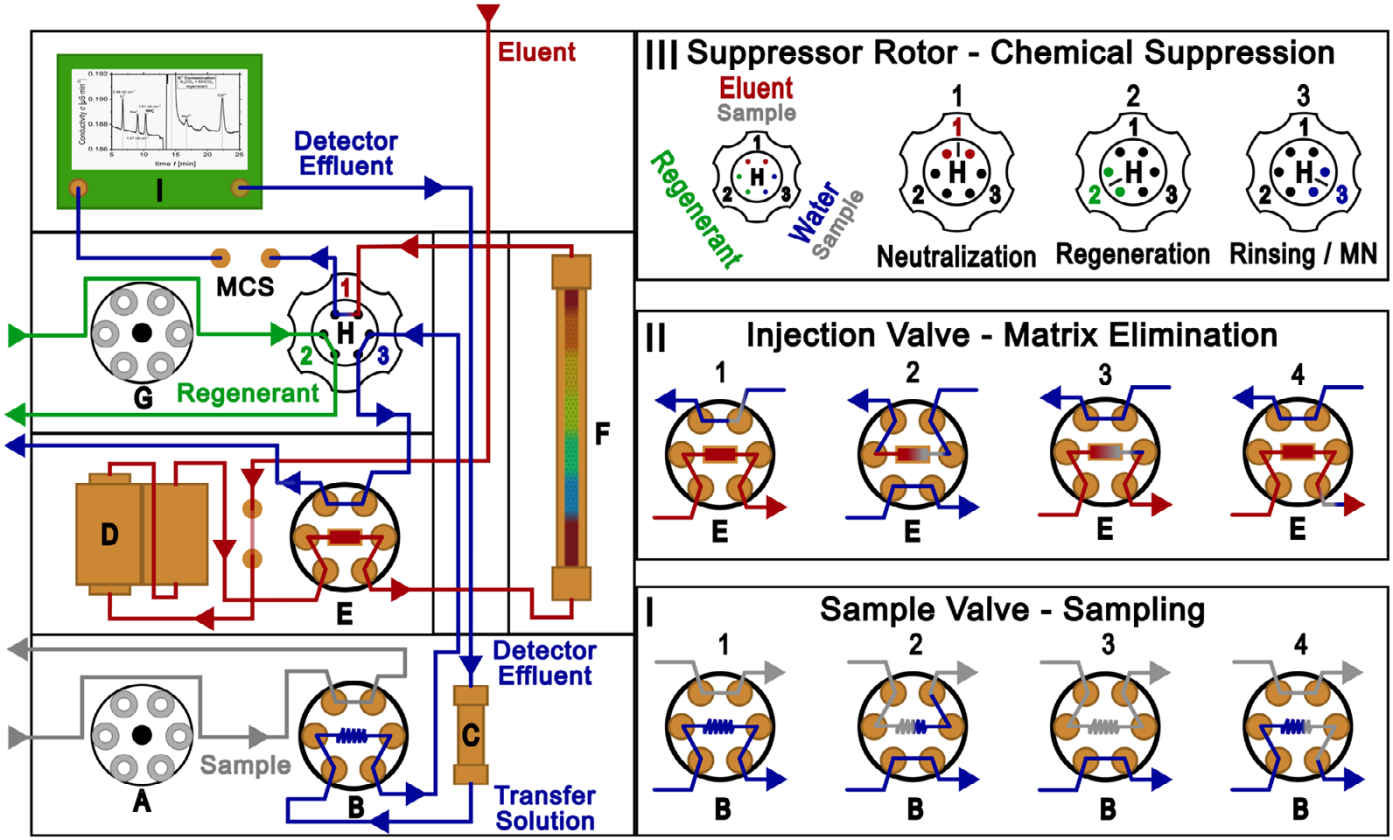
Schematic of the used ion chromatography hardware (left) and central operations (I–III) (right): (A) Peristaltic pump sample, (B) 6‐port sample valve with sample loop, (C) trap column, (D) two‐piston high‐pressure pump, (E) 6‐port injection valve, (F) separation column, (G) peristaltic pump regenerant, (H) suppressor rotor with three WBA resin chambers, and (I) conductivity detector. The fluid paths of the eluent are shown in red, ultrapure water (transfer solution) is shown in blue, the suppressor regenerant is shown in green, and the sample is shown in gray. The sampling at the sample valve (B) with the sample loop is shown on the bottom right (I). The matrix‐elimination (ME) via the enrichment column (E) is shown in the middle on the right (II). Top right (III) shows the chemical suppression of the eluent at the suppressor rotor (H) at position 1 and the matrix neutralization of the sample prior to ME at position 3.

**FIGURE 2:**
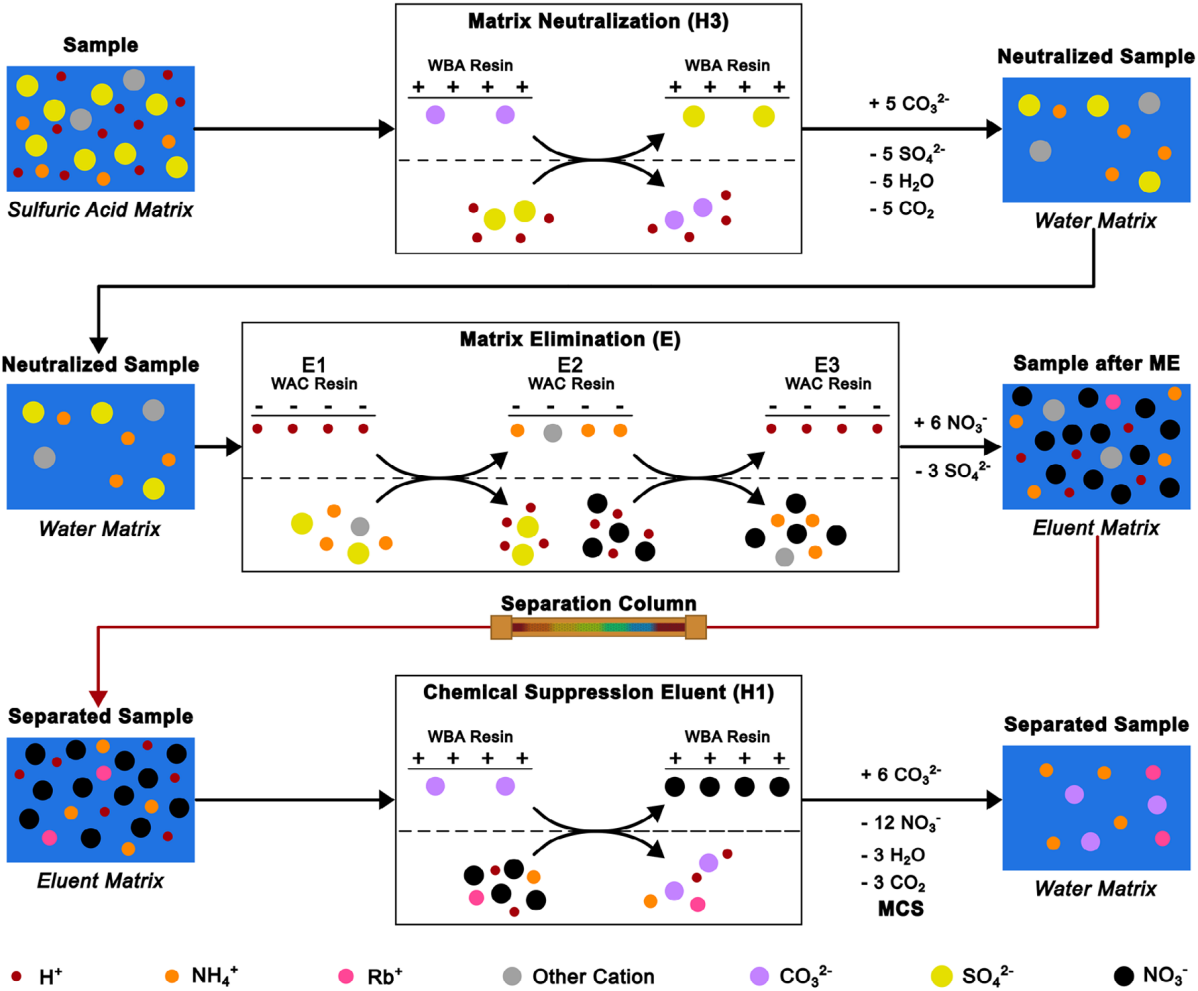
Simplified process depiction of the matrix neutralization (top), matrix elimination (middle), and chemical suppression of the eluent (bottom). The schematically shown ion exchange processes are not balanced with the shown overall sample flow for sake of simplicity. The overall sample flow is balanced for conservation of mass. Both the matrix neutralization and chemical suppression of the eluent occur at the suppressor rotor (Figure 1, H), albeit at different times and in different chambers at position 3 and 1, respectively. The matrix elimination occurs sequentially first by selective retention of the analyte cations ion counter flow and then by elution through the eluent flow (5mM nitric acid with 50 µg L^−1^ Rb^+^).

The starting point for the method development was the need to further improve the detection and quantification of µg L^−1^‐concentrations of ammonium in 0.1 M sulfuric acid electrolyte by IC. While the prior achieved MLOQ of 6 µg L^−1^ ammonium was at the limit of unsuppressed conductivity detection, the necessary manual neutralization of the 0.1 M sulfuric acid electrolyte sample matrix used in our prior work also required a tenfold dilution for sufficient separation and then quantification. The effective MLOQ was in turn increased to 60 µg L^−1^, which was partially compensated for by measuring all samples also spiked with 50 µg L^−1^ lithium and ammonium [8]. Therefore, the best avenue for further improvements was an automated ME. A schematic overview of the ion chromatograph and central operations is shown in Figure 1. The simplified processes of the MN, the ME, and the chemical suppression of the eluent are shown in Figure 2.

Automated ME works by selectively retaining analyte cations on a dedicated enrichment column, which consists of a robust WAC exchange resin (Figure 2, middle). The enrichment column replaces the sample loop at the injection valve (Figure 1, **E**) and is constantly flushed with eluent (Figure 1, **II**, **E1**), except for enrichment with a sample. The sample loop is positioned at the additional sample valve (Figure 1, **B**) at the extension module. The measured‐off sample volume (Figure 1, **I**, **B1–4**) is pushed through the packed bed of the enrichment column in counter flow by the transfer solution, that is, ultrapure water (Figure 1, **II**, **E2**). The analyte cations are selectively retained, while the sample matrix flows on and is discarded (Figure 2, middle). The thus retained and enriched analyte cations are eluted during injection by the constant eluent flow (Figure 1, **II**, **E3,4**) to the separation column (Figure 1, **F**). However, the ME requires a prior MN for strongly acidic samples (pH < 2), because the WAC exchange resin needs a pH above 2 to function. The initially used 0.1 M sulfuric acid electrolyte has a theoretical pH of 0.96 and must be neutralized for the ME. While the acidic sample matrix can be neutralized manually by adding potassium hydroxide to the sample, this introduces high concentrations of undesired potassium into the sample, resulting in a high ionic strength. Although the ME would remove the sulfate of the manually neutralized sulfuric acid sample matrix, the added potassium is retained alongside the analyte cations. The thus introduced potassium is also injected onto the separation column, which seemingly deteriorated the separation column in our prior work possibly due to the high ionic strength [8]. Automated MN prior to ME is achieved by a suppressor column, a dedicated packed bed WBA exchange resin in base form (Figure 1, **H**). The measured‐off sample volume is pushed through the suppressor by the transfer solution and the acid anion of the sample matrix, sulfate, is exchanged for hydroxide or carbonate ions, which neutralize the acid anion (Figure 2, top). The thus neutralized sample matrix enables selective retention of the analyte cations on the WAC enrichment column (Figure 2, middle). Although the use of the suppressor in carbonate/bicarbonate form results in carbonic acid in the sample matrix, alkaline earth metal hydroxides have poor solubilities [9]. This poor solubility causes extensive smearing of alkaline earth metals peaks, if the suppressor is used in hydroxide form, because the counter ions of the analyte cations are exchanged as well. This is especially relevant for suppressed cation chromatography. Furthermore, ammonium (NH_4_^+^_,aq_) is in pH‐dependent equilibrium with dissolved ammonia (NH_3,aq_), which is not detected by a conductivity detector. The ME by enrichment column also results in enrichment of any cationic impurities contained in the system. A varying unknown concentration of inadvertently enriched impurities, such as ammonium, would increase the measured corresponding analyte peak area, which has to be accounted for. The most important source of cationic impurities is the used transfer solution. Although the enrichment of cationic impurities can be compensated for by subtracting a comparable blank measurement that accounts for the same magnitude of impurities, the appropriate used solution is a dedicated strong acid cation (SAC) exchange resin trap column removing cationic impurities from the transfer solution (Figure 1, **C**).

Baseline noise proved substantial for unsuppressed IC at the lowest analyte concentrations in calibration, resulting in insufficient SNR affecting peak detection and peak area quantification [8]. Although chemical suppression of the eluent does not improve detection sensitivity, the greatly reduced baseline conductivity results in a much‐improved SNR. This improvement enables reliable peak detection and quantification at lowest analyte concentration.

However, chemical suppression of the eluent results in noticeable nonlinearity of the calibration curve at low analyte concentrations [9, 10]. The chemical suppression of the eluent was implemented alongside the MN using two chambers of the suppressor rotor (Figure 1, **H**) at once. Metrohm suppressors consist of a PEEK rotor with three dedicated chambers filled with a packed‐bed ion exchange resin. While one chamber is in use to neutralize the eluent at position 1 (Figure 1, **III**, **H1**), the next chamber at position 2 is regenerated (**H2**), while the already regenerated chamber is rinsed at position 3 (**H3**). The continuous operation is realized by rotating the suppressor counterclockwise by 120° per step, so each chamber is used, regenerated and rinsed in turn (**H1‐3**). The chamber at position 2 is regenerated with a mixture of potassium carbonate and potassium hydrogencarbonate. The chamber at position 3 is rinsed using detector effluent (Metrohm STREAM; Suppressor Treatment Reusing Eluent After Measurement) to remove leftover regenerant. This continuous stream of ultrapure water was also used as transfer solution and routed through the lower sample valve (Figure 1, **B**), before passing through position 3 of the suppressor (Figure 1, **H**) and over the enrichment column at the upper injection valve (Figure 1, **E**).

In doing so, we implemented a suppressed cation chromatography with automated MN and ME using one suppressor. A sufficient neutralization capacity per chamber for sample and eluent was ensured by the measurement program.

### 3.2 Chromatogram Standard Cations

**FIGURE 3:**
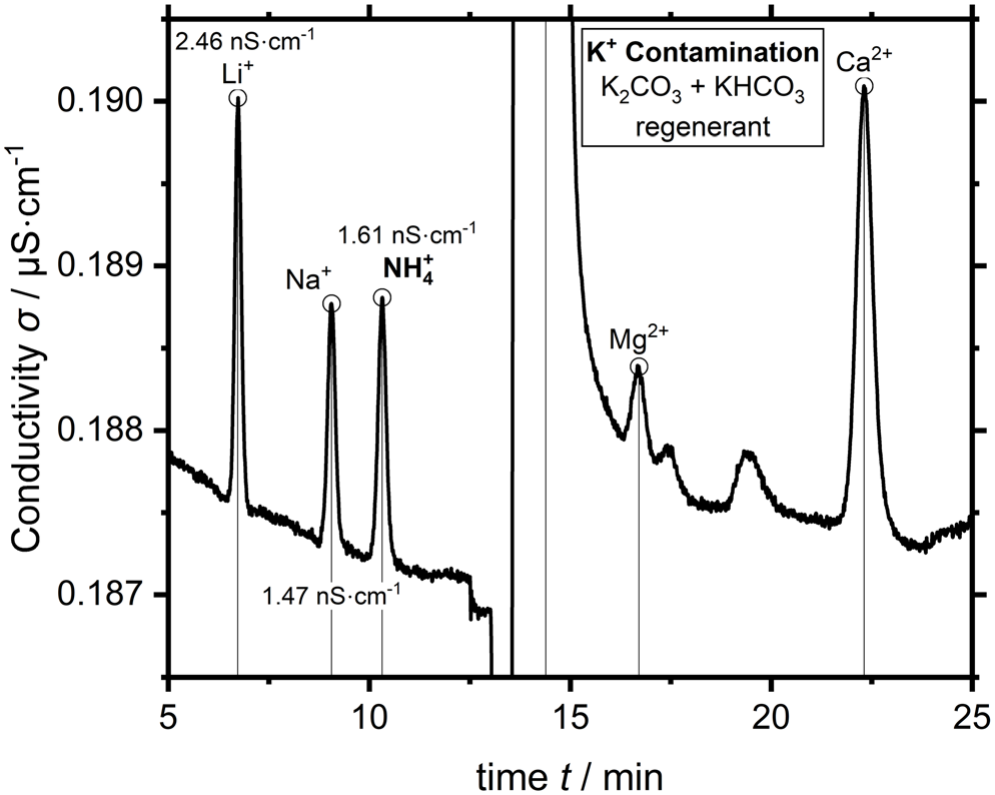
Direct conductivity chromatogram of six standard cations at a concentration of 1 µg L^−1^ (V_inj_ 100 µL) (lithium Li^+^ 6.7 min, sodium Na^+^ 9.1 min, ammonium NH_4_^+^ 10.3 min, Potassium K^+^ 14.4 min, magnesium Mg^2+^ 16.7 min, and calcium Ca^2+^ 22.3 min). Eluent 5 mM HNO_3_ + 50 µg L^−1^ Rb^+^, 0.9 mL min^−1^, column temperature 40°C.

A direct conductivity chromatogram of the standard lithium, sodium, ammonium, potassium, magnesium, and calcium cations at the lowest calibration standard of 1 µg L^−1^ is shown below in Figure 3.

The analyte peaks are well separated and visible above the baseline of ≈0.2 µS cm^−1^ after suppression. The used potassium carbonate regenerant results in a large potassium peak at 14.4 min. Potassium cannot be reliably quantified because of this inherent contamination by the suppressor regenerant. The peak heights are on the order of 1–3 nS cm^−1^ at the analyte concentration of 1 µg L^−1^ and an injection volume of 100 µL. The baseline noise is on the order of <0.05 nS cm^−1^ and the resulting SNR of the analyte peaks is >20. A quantification of analyte concentrations less than 0.2 µg L^−1^ is hypothetically possible based on the commonly accepted SNR limit of 3. However, increasing nonlinearity of the analytical calibration curve becomes relevant at those concentrations. This nonlinearity is caused by a decrease of detection sensitivity and is discussed in the next section. This loss of detection sensitivity necessitates a clean measurement system because even small amounts of contamination will have a large impact in this concentration region. Internal and external ultrapure water blanks were measured to assess the cleanliness of the system. Freshly drawn ultrapure water should not contain relevant concentrations of ions. The internal blank accounts for all system contamination but the autosampler. In contrast, the external fresh ultrapure water blank includes the sampling process and will show possible contamination thereof. The internal blank showed an average ammonium peak area 0.041 ± 0.014 nS cm^−1^·min (empirical standard deviation ESD, *n* = 6). In comparison, the external blank showed a non‐negligible average ammonium peak area of 0.312 ± 0.089 nS cm^−1^·min (ESD, *n* = 6). This small amount of contamination is introduced by the sampling itself, assuming clean ultrapure water. Still, the system has the required cleanliness, using the detector effluent, and a dedicated SAC trap column successfully minimized system contamination.

### 3.3 Analytical Calibration

**FIGURE 4:**
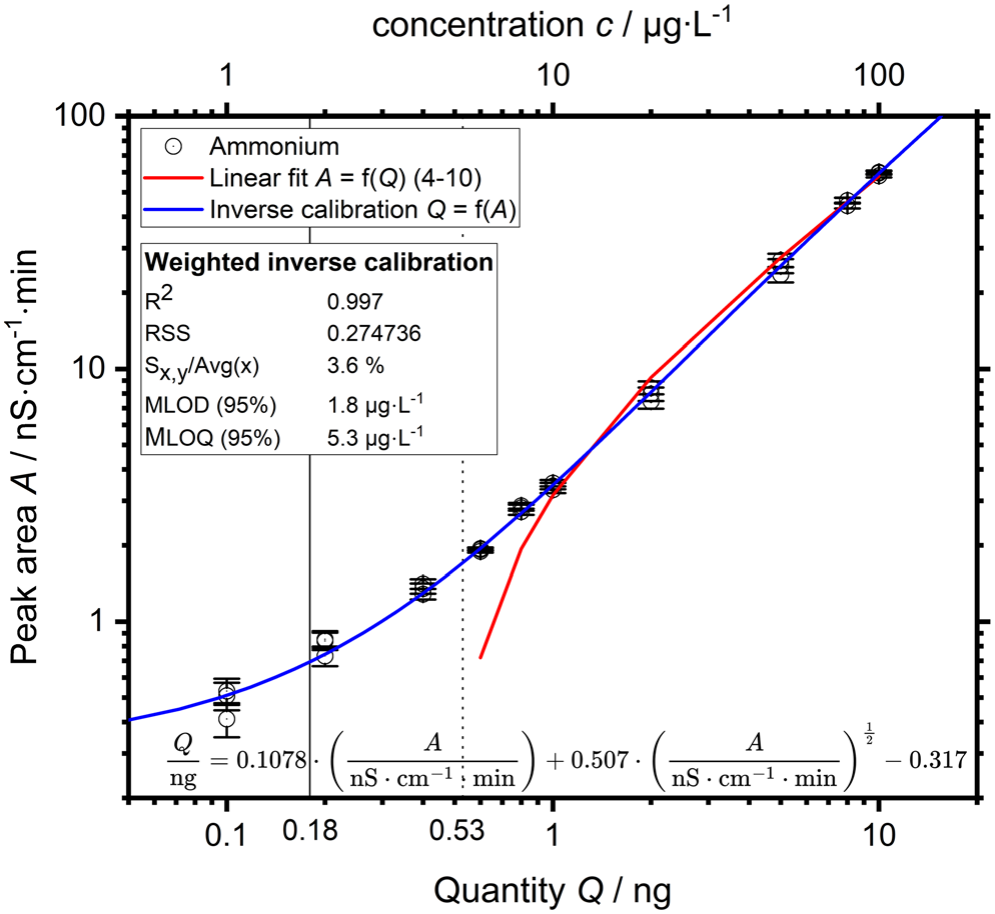
Double‐logarithmic plot of an ammonium calibration from 1 to 100 µg L^−1^ (black circles) with an injection volume of 100 µL. The peak area *A* is the true measure of the injected quantity *Q*, which is shown on the x‐axis. The quantity *Q* is a function of the injection volume *V*_inj_, analyte concentration c and number of enrichments, for which reason it is shown on the x‐axis instead of the concentration. The concentration *c* is shown on the second x‐axis at the top. The error bars show the empirical standard deviation (*n* = 3). A linear fit of the standards from 0.6 to 10 ng shows the increasing nonlinearity below 1 ng analyte.

The EAS production rates to date are low, mostly on the order of a few pmol s^−1^ (<1 µg h^−1^) depending on the catalyst and experimental conditions. An increased experimental duration can compensate this only partially. The analyte concentrations are consequently low as well, on the order of µg L^−1^ ammonium, depending on the electrolyte volume. A considerably large calibration range is still required because of a wide concentration range as a result of contamination. Figure 4 shows a calibration of ammonium from 1 to 100 µg L^−1^ as an example. The analyte solutions were acidified with nitric acid to a concentration of 0.1 wt.‐% identical to commercial standards. Each concentration was measured three times, the empirical standard deviation is shown at each individual measurement point.

Figure 4 shows the measured peak area *A* in nS cm^−1^ min as a function of the standard analyte quantity *Q* in ng, the peak area is the true measure of the analyte quantity in case of a conductivity detector [11]. The double‐logarithmic plot shows the increasing nonlinearity below an analyte quantity of 1 ng, that is, 10 µg L^−1^ at an injection volume of 100 µL. The auto‐dissociation of water becomes relevant at low analyte concentrations, resulting in a non‐constant background conductivity, which causes non‐linearity and in turn much lower detection sensitivity in this region. Ionic impurities affect this non‐linearity and removal of dissolved CO_2_ is essential [10]. Rubidium nitrate (50 µg L^−1^ Rb^+^) was deliberately added to the eluent as an ionic impurity to improve non‐linearity [12] and bind remaining CO_2_ as recommended by the manufacturer. A quadratic fitting function is recommended for suppressed IC because of the aforementioned non‐linearity [10]. Lower and higher order fitting functions were tested. The residual sum of squares (RSS) and predicted residual error sum of squares (PRESS) were used for selection of a fitting function. A higher order fitting function with a lower RSS value was only chosen if the PRESS value was on the same order of magnitude or lower than the PRESS value of the lower‐order fitting function. An inverse calibration, that is, fitting *Q* against *A*, was also tested [13]. Adding a square root term as an educated guess had a good result in this instance. The method limit of detection (MLOD) calculated from this calibration function was 1.8 µg L^−1^ ammonium (Figure 4), which is only slightly lower than the 2 µg L^−1^ in our prior work. A weighted inverse calibration up to 1000 µg L^−1^ resulted in an almost identical MLOD (see Supporting Information).

To sum up, although the direct conductivity detection by chemical suppression of the eluent greatly improves the SNR and in turn enables reliable peak quantification at these low concentrations, the non‐linearity at low concentration results in a reduced detection sensitivity and a larger impact of at these concentrations ubiquitous contamination. Nonetheless, the relative standard error of the calibration curve is low with 3.6%. Further improvements of the MLOD are most feasibly achieved by replicate sample measurements. A single replicate sample measurement improves the MLOD from 1.8 to 1.3 µg L^−1^, that is, a 28% reduction. The next section covers the practical application of the developed method for the determination of relevant background contamination of a selection of used materials and in the used electrochemical measurement cell by online measurement.

### 3.4 Practical Application

eNRR research to date solely relies on the quantitative determination of the electrochemically produced ammonia to determine activity and selectivity of tested catalyst materials. However, low production rates are aggravated by ubiquitous low‐ and high‐intensity sources of environmental contamination. For this reason, quantitative trace analysis is crucial. Quantifying potentially relevant sources of contamination is paramount, because not all sources of contamination can feasibly be removed or avoided.

### 3.5 Background Contamination of Used Materials

The materials used in EC NRR experiments can be unknown sources of contamination as reported in literature [5, 14, 15]. Therefore, different used materials were aged in 50 mL of 0.2 M sulfuric acid electrolyte for 24 h to examine possible cationic contamination introduced by these materials. The aged electrolyte solution was subsequently analyzed by IC (Table 1). The aged electrolyte without any added material was measured as a comparison baseline. A Nafion NR212 membrane was measured as the standard proton exchange membrane material. It was measured pristine (pretreated) and cleaned as described by Hanifpour et al. [14]. The pristine gas diffusion layer (GDL) material Freudenberg H2315 I2 C6 was measured as basis for the catalyst‐coated GDL, that is, the GDE used in experiments. The synthesized ZrN catalyst powder [16] was separately analyzed for chemical dissolution, providing a baseline for noncatalytic production of ammonium. Additionally, the in‐house nitrogen 5.0 quality gas supply was analyzed for ammonia contamination using gas washing bottles filled with 0.2 M sulfuric acid.

**TABLE 1:** Background contamination of used materials as measured in 0.2 M sulfuric acid electrolyte after 24 h aging.

Sample	**Concentration / µg L** ^**−1**^		
	**Sodium Na** ^**+**^	**Ammonium NH**_**4**_^**+**^	**Calcium Ca** ^**2+**^
Electrolyte	2.4	6.6	16.2
NR212, pristine^a^	68.8	15	738.1
NR212, cleaned^b^	23.6	6.2	863.2
GDL, pristine^c^	5.4	4.9	16.5
GDE, ZrN^d^	7.3	35.9	40.4
ZrN catalyst^e^	—	45.1	—
Nitrogen 5.0^f^	—	14.8	—
Nitrogen 5.0, purified^g^	—	<MLOD	—

a Boiled in 3% H_2_O_2_, ultrapure water and 0.1 M H_2_SO_4_ each for 1 h, rinsed and stored in water.

b Cleaned using the protocol by Hanifpour et al. immediately before use of the membrane.

c Freudenberg H2315 I2 C6, batch WE 738–10.0.13.

d GDL coated with 0.95 mg cm^−2^ ZrN, containing 10.4 wt.‐% Nafion (D2021) in the catalyst layer.

e Carbothermal nitridation ZrN C2.2, 15 mg, 50 mL, 2.25 ± 0.03 µg ammonium total, *n* = 3.

f Nitrogen 5.0, 10 mL min^−1^, 21.53 h, 12.92 L total, 0.2 M H_2_SO_4_, 50 mL, 80 ppb_mol_, 0.55 pmol s^−1^.

g Agilent OT3‐4 [5], 10 mL min^−1^, 64.32 h, 38.57 L total, 0.2 M H_2_SO_4_, 50 mL, 3 ppb_mol_, <0.05 pmol s^−1^.

The 24 h aged electrolyte prepared from ultrapure 96% sulfuric acid stock contained negligible concentrations of sodium Na^+^, ammonium NH_4_^+^, and calcium Ca^2+^ ions. The pristine Nafion membrane showed minor NH_4_^+^ contamination, some Na^+^ contamination, and unexpectedly an order of magnitude larger Ca^2+^ contamination. While the NH_4_^+^ contamination is reduced to the electrolyte baseline by the dedicated cleaning procedure [14], some Na^+^ contamination remains. By contrast, the Ca^2+^ contamination is increased after cleaning. The high Ca^2+^ contamination is unique to the Nafion membrane within these samples. This points to an inherent contamination of the Nafion membrane material with moderate Na^+^ and high Ca^2+^ concentrations. The pristine GDL shows no contamination compared to the electrolyte. In contrast, the GDE shows some NH_4_^+^ and Ca^2+^ contamination. The total amount of NH_4_^+^ liberated from the GDE accounting for the GDL is 1.55 µg. This equals 1.03 µg cm^−2^ (1.51 cm^2^ sample) or 0.66 pmol cm^−2^ s^−1^ per 24 h. Chemical dissolution of the ZrN catalyst accounts only for 12.1% of this (0.08 pmol mg^−1^ s^−1^ NH_4_^+^, see Supporting Information), which is comparable to literature [17]. While the liberated contamination accounts only for about 4% of the ion exchange capacity (IEC) in case of the NR212 membrane, it accounts for 61.6% of the IEC of the ionomer inside the catalyst layer of the GDE. Paradoxically, the ionomer inside the catalyst layer seems to contain and release more contamination than the Nafion membrane material itself, which will be discussed in detail in the discussion section.

### 3.6 In Situ Background Contamination

Although the developed analytical method is a powerful tool for the trace quantification of ammonium and useful for the application, manual sampling and the required sample volume curtail deeper insights in the dynamics of eNRR. Use of the autosampler requires a minimum sample volume of 2 mL, of which only 100 µL are injected into the separation column as sample. The electrolyte volume in the measurement cell is only about 35 mL, of which even less can be safely sampled. Furthermore, manual sampling limits the number of samples and the frequency of sampling. More importantly, manual sampling is a possible entrance for contamination.

**FIGURE 5:**
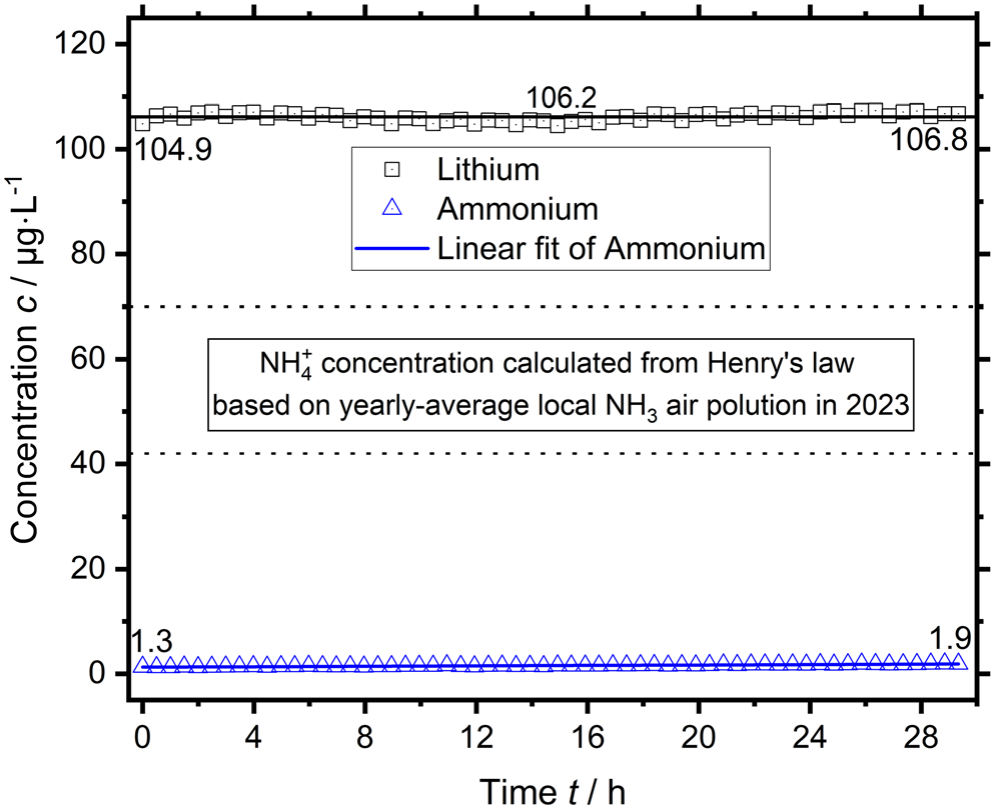
Online IC measurement of freshly prepared 0.2 M sulfuric acid electrolyte spiked with 100 µg L^−1^ Lithium in a 100 mL volumetric flask.

For these reasons, a basic direct online coupling of the electrochemical cell setup with the ion chromatograph was tested. The electrolyte was circulated continuously through the 100 µL sample loop by a peristaltic pump. This form of liquid delivery and circulation was tested separately and had no noticeable detrimental effect on analyte carryover. However, repeated measurement with circulation results in a progressive dilution of the circulated volume because the sampled volume of 100 µL is replaced with ultrapure water, which has to be accounted for. To scope the possible ingress of environmental ammonia contamination and test the online ammonium measurement by IC, freshly prepared 0.2 M sulfuric acid spiked with 100 µg L^−1^ Li^+^ inside a 100 mL volumetric flask open to the atmosphere was circulated and measured. The measurement is shown in Figure 5.

The measured Li^+^ concentration is constant over time accounting for dilution. The average measured concentration of 106.2 µg L^−1^ is consistent with the spike concentration, there is no loss of Li^+^ over time. The measured NH_4_^+^ concentration shows a small linear increase from 1.3 to 1.9 µg L^−1^, but is overall below the MLOD. An increasing NH_4_^+^ concentration is anticipated for ingress of environmental ammonia contamination, but is below the expected magnitude (see Supporting Information). Prior results had shown an ingress of environmental ammonia contamination into open vessel water blanks consistent with the concentration range calculated from the geographically reported annual‐average ammonia air pollution [8]. A possible explanation is a seasonally negligible environmental contamination in the winter of 2024. The additional diffusion resistance of the bottleneck cannot explain the nearly constant ammonium concentration, because the measurement duration of more than 28 h should be sufficient to compensate for that. The online IC measurement was successfully shown and there was negligible ingress of environmental ammonia contamination in this case for a 100 mL volumetric flask.

**FIGURE 6:**
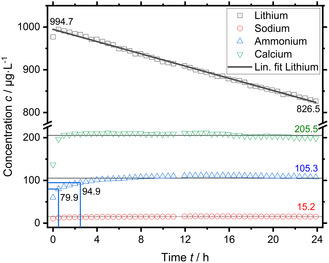
In situ background contamination measurement of the electrochemical setup with an already tested ZrN‐GDE by online IC using 0.2 M sulfuric acid electrolyte accidentally spiked with 1000 µg L^−1^ instead of 100 µg L^−1^ Li^+^. The concentrations 79.9 and 94.9 µg L^−1^ at 0.5 and 2.5 h, respectively, are marked, which coincide timewise with the sampling in a turnover‐experiment.

The **in situ background contamination** was further investigated by measuring freshly prepared electrolyte in the electrochemical measurement cell with a mounted GDE, but without any electrochemistry. The EC setup was identical to an actual electrochemical measurement except for a reference electrode or electrical connections, that is, at open‐circuit voltage (OCV). An already electrochemically tested GDE sample was deliberately chosen to start with, because the production rate had already been determined in a turnover‐experiment. The electrolyte used in this experiment was accidentally prepared with a spike concentration of 1000 µg L^−1^ instead of the intended 100 µg L^−1^ Li^+^. The measurement is shown in Figure 6.

**FIGURE 7:**
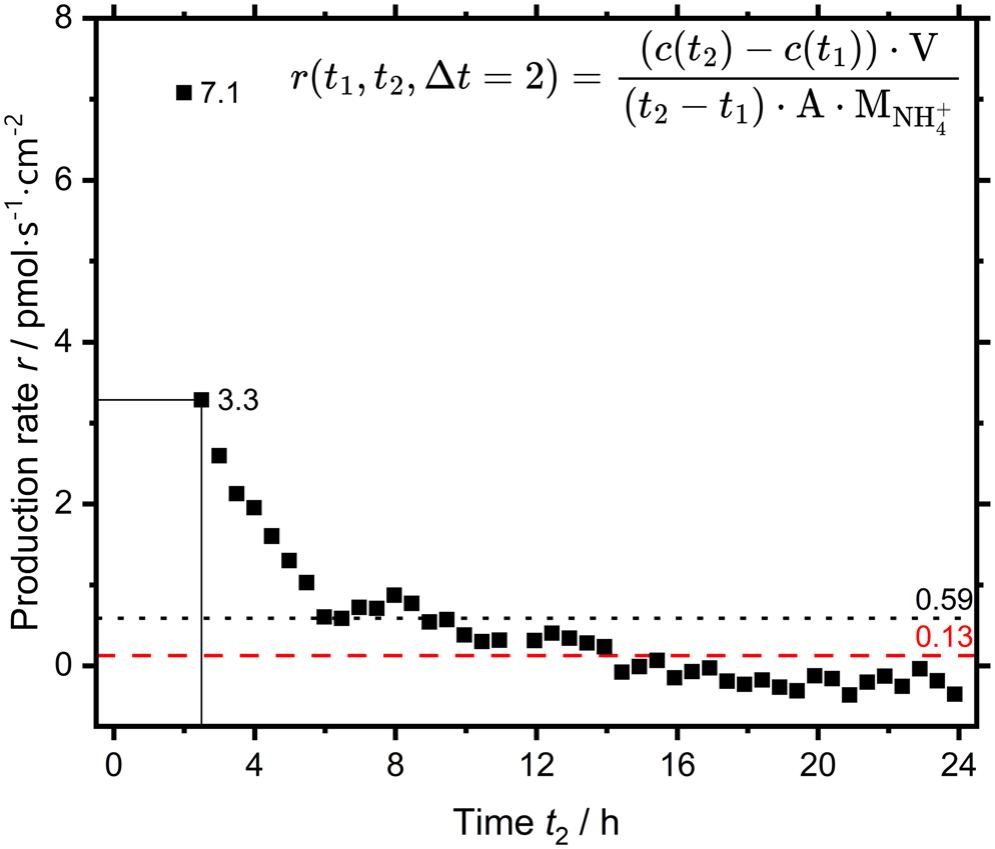
Rate of change of the ammonium concentration calculated for a 2 h window from the time‐resolved concentration data. The marked production rate of 3.3 pmol s^−1^ cm^−1^ is derived in a turnover‐experiment by taking a sample at 0.5 and 2 h, that is, start and finish of the constant potential block as done in our protocol.

In contrast to the prior measurement, there is a significant NH_4_^+^ concentration, as well as Ca^2+^ and to a lesser extent Na^+^. While Li^+^ shows a significant linear downwards trend, NH_4_^+^ and Ca^2+^ show a faster asymptotic trend typical for an equilibrium process. Although the time resolution of one measurement approximately every 30 min cannot resolve such a trend for Na^+^, it is implied. The time‐weighted average NH_4_^+^ concentration of 105.3 µg L^−1^ is comparable to the EC measurement of this GDE sample (see Supporting Information) and literature references [15]. While Ca^2+^ had been found alongside NH_4_^+^ in EC measurements, the concentration was significantly lower, and no closer attention was given, because trace level Ca^2+^ concentrations are regularly observed. Due to a similar trend in concentration, a common contamination source is assumed for NH_4_^+^, Ca^2+^, and Na^+^. A significant Ca^2+^ contamination has been shown for the Nafion NR212 membrane (Table 1). Therefore, the Nafion ionomer content of the GDE is again the most likely source. Fabrication of GDEs without Nafion ionomer had been tried because of just such potential contamination, but was not feasible. The catalyst layer without Nafion ionomer was not water‐resistant, it disintegrated upon contact with water. Subsequently, the Nafion ionomer inside the catalyst layer had been disregarded as significant source of contamination based on the order of magnitude lower IEC compared with a used Nafion membrane and the negligible contamination found within such membrane [18]. However, even though the net change in NH_4_^+^ concentration during a 2 h constant potential was used in EC turnover‐experiments to determine the production rate, the here shown dynamic of the NH_4_^+^ concentration in the first 6 h is relevant. Figure 7 shows the rate of change of the NH_4_^+^ calculated for a 2‐h window, the length of EC turnover‐experiments used at a set EC potential.

**FIGURE 8:**
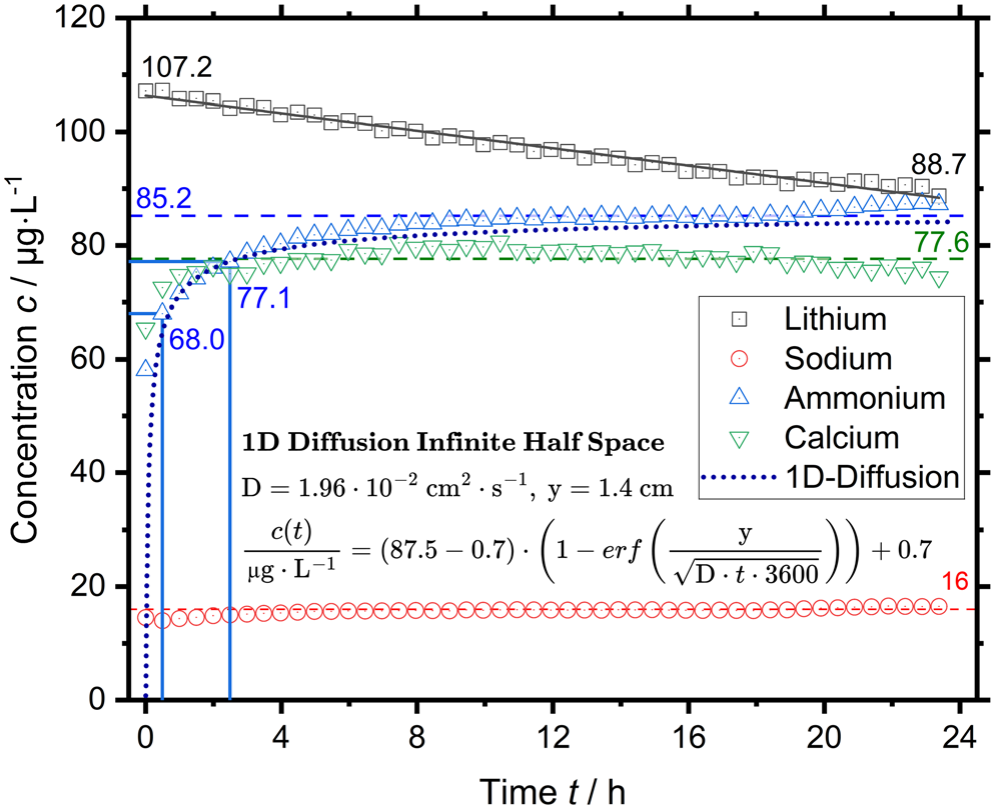
In situ background contamination measurement of the electrochemical setup with a pristine ZrN‐GDE of the same batch by online IC using 0.2 M sulfuric acid electrolyte spiked with 100 µg L^−1^ lithium. The ammonium concentration has been fitted with the analytical solution to 1D‐diffusion into an infinite half‐space by adjusting the distance y.

Although the comparatively high initial rate of 7.1 pmol s^−1^ cm^−2^ levels off within half an hour to 3.3 pmol s^−1^ cm^−2^, this is still of relevant magnitude compared to prior determined production rates of 1–3 pmol s^−1^ cm^−2^. The same holds true for the average of 0.59 pmol s^−1^ cm^−2^, but not the time‐weighted average of 0.13 pmol s^−1^ cm^−2^, which is negligible. This form of contamination would result in a false‐positive production rate of 3.3 pmol s^−1^ cm^−2^ assuming the same behavior in EC turnover‐experiments and matches prior results. While the contamination issue of Nafion is well known in literature, it was underestimated for the Nafion ionomer of the catalyst layer. However, the found ammonium might have originated in the prior EC turnover‐experiment of the used GDE sample. To this end, the measurement was repeated with a pristine GDE sample of the same batch, which is shown in Figure 8.

The pristine GDE shows an almost identical result to the tested GDE sample of the same batch, significant concentrations of NH_4_^+^ and Ca^2+^ and to a lesser extent Na^+^ are found. The spiked Li^+^ concentration shows a linear downwards trend, whereas, the NH_4_^+^ and Ca^2+^ concentration quickly increase, reaching asymptotic time‐weighted average values of 85.2 (2.76 µg) and 77.6 µg L^−1^, respectively. Even though the Ca^2+^ concentration is less than half compared to the used GDE sample, the NH_4_^+^ concentration is comparable to the used GDE sample and again of similar level to EC turnover‐experiments. Moreover, the Na^+^ concentration is almost identical.

While the 2‐h‐window ammonium release rate calculated for the pristine GDE sample is lower, the value of 1.98 pmol s^−1^ cm^−2^ at 2.5 h matches prior production rates determined in turnover‐experiments as well, again pointing to prior false‐positive results caused by a previously judged negligible source of contamination. If the Nafion ionomer inside the catalyst layer is the source of contamination, then the concentration profile should match an applicable description of the diffusion process reasonably well. The solution to one‐dimensional diffusion from a plane into an infinite half space [19] fits the NH_4_^+^ concentration as function of time well with an average deviation of less than 5%, explaining the observed results. The mismatch at the beginning and the remaining offset are likely due to insufficient time resolution for the rapid change in concentration at the beginning, and in reality, more complex multicomponent diffusion of the cations out (Na^+^, NH_4_^+^, Ca^2+^) and into (H^+^, Li^+^) the ionomer. The determined concentrations of NH_4_^+^, Ca^2+^, and Na^+^ account for 98% of the calculated IEC of the ionomer inside the catalyst layer.

The Nafion dispersion (D2021) used for hand‐spraying of GDEs was analyzed for ionic impurities by IC in order to determine whether the found contamination is contained within the material from the start. The 20 wt.‐% dispersion was diluted to the concentration used in hand‐spraying (see Supporting Information). The found NH_4_^+^ concentration was below the MLOQ of 5.5 µg L^−1^. The Nafion dispersion does not contain significant NH_4_^+^ or cationic contamination. If the Nafion inside the catalyst layer of the GDE is the source of the found NH_4_^+^ contamination, it is a secondary source, that is, the initially clean Nafion retained ammonia contamination from contact with an environmental source. However, the manufactured GDEs were specifically stored in a nitrogen filled desiccator to prevent this. The possible uptake of minute ammonia contamination in the used nitrogen gas supply as cause is treated theoretically in the following discussion.

## 4 Discussion

The developed suppressed IC method with automated MN and ME enables trace analysis of less than 1–1000 µg L^−1^ ammonium. Although the chemical suppression decreases the inherent cation detection sensitivity, the greatly improved SNR makes reliable quantitative detection of sub‐ppb concentrations possible. Having said this, auto‐dissociation of water causes further loss of detection sensitivity at low analyte quantities, below 1 nmol depending on analyte, and in consequence, a nonlinear analytical calibration curve. The cation detection sensitivity may be improved by switching from the suppression with CO_3_^2−^/HCO_3_^−^ to OH^−^. However, peak tailing would be adversely affected. This is especially the case for the alkaline earth metal cations Mg^2+^ and Ca^2+^. The resulting carryover of present Mg^2+^‐ and Ca^2+^‐traces from one chromatogram to the next would affect peak quantification. Detection of these other cations along NH_4_^+^ is an advantage of IC. Furthermore, in case of NH_4_^+^, some of the increased detection sensitivity gained would be lost to the pH‐dependent equilibrium between ammonium hydroxide and undetected dissolved ammonia. The nonlinearity inherent to suppressed conductivity detection at low analyte concentrations can be improved further by adjusting the amount of added ionic impurity (Rb^+^) as described by Eom et al. among other things [10, 12]. Adjusting the amount of added ionic impurity is a delicate tradeoff between better linearity and in consequence of the increased baseline worsened SNR [12]. Neglecting the impact of CO_2_ and other impurities, slightly increasing the Rb^+^ concentration from 50 to 85 µg L^−1^ would theoretically reduce nonlinearity significantly for NH_4_^+^ by suppressing the effect of water dissociation further. Although the developed suppressed IC method hitherto only slightly improved the MLOD from 6 µg L^−1^ in our prior work to 5.4 µg L^−1^ in this work, the system is superior in reliability and purity as indicated by a negligible ammonium peak area in internal blanks of less than 0.05 nS cm^−1^ min. Furthermore, the ME also enables sample enrichment, which allows to quantify samples below MLOQ by deliberate enrichment. Especially the online in situ IC measurements greatly enhance the capabilities as show by the exemplary application.

Although the method was developed for the quantitative trace analysis of ammonium in a strongly acidic sample matrix, other matrix compositions should work as well within limits. In general, sample compositions within the material compatibility of directly contacted columns (suppressor, enrichment column, 1 ≤ pH ≤ 14) and operational window of the ion exchange processes can be analyzed. Samples with added organic modifiers such as alcohols, often used as additives in photocatalytic research, can be analyzed directly using ME. Samples with a high pH or high ionic strength are on one hand constrained by sufficient competitive retention of analyte cations on the enrichment column, which depends on the IEC and selectivity of the enrichment column, and on the other hand by the large concentration difference of analyte and matrix cations, that is, sufficient separation efficiency for analysis. A sufficient separation efficiency is necessary to separate the cations on the separation column into well resolved individual peaks for detection and quantification. The application dependent choice of separation column is central for the separation efficiency. We therefore specifically chose a high‐capacity separation column with excellent resolution of sodium Na^+^ to ammonium NH_4_^+^ [20], which elute closely. Provided both sufficient retention and separation applies, high‐pH samples can be analyzed. However, ammonia is a weak base, contained in a high‐pH matrix as a dissolved gas with low solubility in water. The loss of ammonium as outgassing ammonia likely renders direct analysis of high‐pH samples moot. Dedicated acid traps are commonly used to retain (outgassing) ammonium in acidic acceptor solutions [15]. Insufficient separation efficiency in case of high ionic strength can be addressed by adequate sample dilution, assuming sufficient analyte concentration after dilution [8]. That being said, the outgassing of ammonia at high pH can be utilized on purpose to transfer ammonium (ammonia) as analyte from a complex sample matrix to a suitable acceptor solution, eliminating the high ionic strength sample matrix. This is known as purge‐and‐trap and has been demonstrated in literature for seawater using an inert carrier gas [21]. Dialysis can similarly be employed for ammonium using a membrane contactor as demonstrated by Hanifpour et al. for fluorescence analysis of ammonium [14]. Both methods must be included in the calibration to account for the collection efficiency of the transfer process. The developed method can therefore be used for a broad range of samples and applications.

The developed IC method was applied to determine the background contamination offline and online. The offline investigation of a limited number of relevant materials as expected showed some background contamination. Contamination of the SAC exchange material ionomer Nafion is well known [14]. The determined amount of NH_4_^+^ contamination is low compared to literature. The degree of NH_4_^+^ contamination varies and is likely influenced by the geographical and local environment as well as storage. The investigated Nafion membrane sample was typically prepared for Nafion, that is, boiled in hydrogen peroxide, sulfuric acid, and ultrapure water [14], which likely removes most contamination. Additionally, the thus prepared membrane was stored in ultrapure water in a closed container, which likely limited reuptake of environmental NH_3_ contamination. The order of magnitude larger Ca^2+^‐contamination was unexpected, but is explained by the high selectivity of Nafion for Ca^2+^. Interestingly, this is not mirrored in the investigated GDE samples, which contain Nafion in the catalyst layer. Therefore, the Nafion membrane material is inherently contaminated with Ca^2+^. The GDE sample showed some NH_4_^+^ contamination, which is not present in the GDL material used for manufacturing the GDEs. The separately measured chemical dissolution of the ZrN nitride catalyst does not fully account for the NH_4_^+^ contamination of the GDE. Thus, it must originate in the Nafion inside the catalyst layer of the GDE sample. The subsequently possibly measured production rate of 0.66 pmol cm^−2^ s^−1^ is non‐negligible, but is not sufficient for the found in situ background contamination of the EC setup.

The in situ online IC measurement at OCV established a rapid liberation of a significant ionic contamination by the GDE sample. The online measurement was able to resolve the time evolution, which elucidated a likely false‐positive production rate in prior EC turnover‐experiments. The liberation is rapid and reaches equilibrium within 4–6 h in the investigated system and conditions. Nonetheless, when the concentrations are coincidentally measured following protocol after 0.5 h and 2.5 h in EC turnover‐experiments, this concentration profile results in a false‐positive production rate of about 2–3 pmol cm^−2^ s^−1^ in this instance. Although the magnitude varied between the used and pristine GDE sample, the overall behavior was independent of the sample history. Because neither chemical dissolution of the nitrogen‐containing ZrN catalyst nor inherent contamination of the Nafion dispersion or GDL used for the GDE manufacture can explain the found contamination. The Nafion contained in the catalyst layer must be a secondary source of significant contamination. The Nafion contained in the catalyst layer had hitherto been judged negligible based on the IEC and magnitude of NH_4_^+^ contamination found inside Nafion membrane material. However, the Nafion‐containing catalyst layer seems to retain and emit more NH_4_^+^ contamination than the membrane. This is illustrated by the quantified contamination as percentage of the IEC, which is only 4% for the tested membrane, whereas it is almost 70% for the tested GDE after 24 h aging in electrolyte and 98% for the tested pristine GDE in the second in situ measurement.

The hand‐sprayed GDEs were deliberately stored in a nitrogen‐purged desiccator to prevent contact and uptake of environmental contamination. The nitrogen supply was of 5.0 quality, which contained an ammonia contamination of only 80 ppb_mol_ (see Supoorting Information). In comparison, the geographical average annual environmental ammonia contamination was 11 ppb_mol_ (7.7 µg m^−3^) in 2023 [22]. This contamination of the nitrogen gas supply is reduced below IC MLOD by use of an Agilent OT3−4 gas purifier, as tested by Izelaar et al. [5]. The nevertheless calculated remaining ammonia contamination is below 3 ppb_mol_. Nonetheless, even continuous uptake of ammonia from a local low impact source can eventually saturate the Nafion inside the GDE catalyst layer, if no prior equilibrium is reached. The equilibrium ammonium uptake of Nafion is a function of the IEC and ammonium and proton concentration in solution [23]. Although Nafion as a SAC ionomer has a high selectivity for cations over protons [24], the theoretical pH of 0.68 of the electrolyte, 0.2 M sulfuric acid, is sufficiently high to fully protonate the Nafion and liberate the contained cationic contamination at the found µg L^−1^‐concentrations (see Supprting Information). The inverse, a small ammonium concentration at a moderate pH, readily explains the uptake of ammonia by the initially clean Nafion dispersion inside the GDE. The determined ammonia contamination of 80 ppb_mol_ of the used nitrogen 5.0 house gas supply theoretically results in an equilibrium ammonium ion content of 0.89 of the Nafion inside the GDE catalyst layer based on Henry's law and the data by Hongsirikarn [23], assuming the Nafion retains some water as initial reservoir for the ingress of ammonia (see Supporting Information). The resulting ammonium concentration in the electrolyte calculated after liberation is roughly 100 µg L^−1^ (see Supporting Information), which matches the measured concentrations of 85.2 and 105.3 µg L^−1^ reasonably well (Figures 7 and 8). This highly simplified calculation shows that any non‐negligible environmental ammonia contamination at a ppb‐level will saturate Nafion to a large degree due to the high selectivity (see Supporting Information). The high surface area of the GDE is beneficial for these exchange processes. To summarize, even though the nitrogen 5.0 gas supply ammonia contamination is only 80 ppb_mol_ and the resulting equilibrium ammonium concentration in solution is similarly low, the moderately high pH of the assumed retained ultrapure water causes a high equilibrium ammonium ion content of the Nafion inside the GDE catalyst layer, which is then liberated by the electrolyte. If this explanation holds true, the reason for the in relation much lower ammonium contamination of the Nafion membrane material is the storage, not the inherent material behavior, as hypothesized. Consequently, Nafion‐containing GDEs have to be deliberately cleaned similarly to Nafion membranes, if possible. The effect of such a cleaning procedure on the GDE as a whole will have to be considered. Apart from removing contamination, resolving the time‐evolution of the ammonium concentration in actual electrochemical turnover‐experiments by online IC will enable direct measurement of actual production rates.

## 5 Conclusion and Outlook

The possibilities of EAS as prospective direct electrochemical synthesis of ammonia using renewable energy have garnered great interest in the field. Research on EAS by eNRR is still solely reliant on the quantitative determination of produced ammonia (ammonium) for assessing novel catalysts. The reported production rates to date are low, necessitating quantitative trace analysis, which also aggravates the impact of ubiquitous low and high‐intensity sources of contamination. Preventing contamination is paramount but only to a degree feasible and practical. Locating and assessing unavoidable contamination is equally important. Furthermore, differentiation between genuine catalytic EAS and a limited or complete dissolution of a tested novel catalyst during EC testing is of high interest to improve understanding of the material behavior specifically and the eNRR in general. Determining the liberation of ammonium by chemical dissolution of the catalyst over a certain duration yields an upper limit for noncatalytic ammonium production. Nevertheless, investigating the chemical catalyst dissolution over time will deepen the material understanding, allowing to select for beneficial material properties.

Determining the ammonium concentration in situ over time in EC eNRR experiments using online IC will not only allow to directly determine ammonium production rates, but will also enable to link the effect of the applied EC potential and other key process parameters, for example, dynamic cycling, degradation, process temperature, or gas flow rate. Thus, the next step in our work will be to integrate EC and online IC, that is, pairing a suitable EC potentiostat and the IC. This will allow to record chromatograms at set points in the EC sequence, for example, before and after a potential step. All this is possible because of the developed analytical IC method, which enables reliable ammonium quantification at µg L^−1^‐concentrations directly in 0.2 M sulfuric acid electrolyte. Further improvements might be achieved by optimizing the chemical suppression and the eluent composition, albeit at a tradeoff as described. More expedient is improving the chromatogram duration, which limits the total measurement duration to 30 min and thus directly the time resolution. The chromatogram duration can feasibly be reduced by employing a specifically designed eluent gradient or possibly by excluding alkaline earth metals and stopping the chromatogram after potassium. Both can potentially improve the time resolution to 20 min, it is however challenging to design a suitable eluent gradient that optimizes duration without loss of resolution. A different attractive addition to the IC would be UV/Vis spectroscopy, to simultaneously quantify nitrate and nitrite contamination in samples. Nitrate and nitrite are common forms of nitrogen contamination, which are more easily reduced to ammonium than elemental nitrogen. Thus, nitrate and nitrite contamination are common sources for false‐positive results as well. While nitrate is directly spectroscopically determined, simultaneous determination of nitrite alongside nitrate has been shown by second‐derivative UV/vis spectroscopy [25]. A comprehensive quantitative determination of ammonium, nitrate, and nitrite within one analytical method would improve the analytical capabilities greatly.

To conclude, the developed suppressed IC method enables reliable quantification of ammonium at ppb concentrations and online IC measurements are able to resolve the time evolution. The online IC measurement elucidated the Nafion ionomer content of tested GDE samples as source of relevant ammonium contamination, which was hitherto considered negligible. The rapid liberation of this contamination upon contact with the electrolyte likely resulted in prior false‐positive production rates. The different behavior of the Nafion ionomer in the catalyst layer compared to Nafion membrane material is theoretically explained by continuous uptake of low intensity local ammonia contamination in the atmosphere. Future EC turnover‐experiments directly coupled with online IC will ensure even more reliable experimental investigation of catalysts for EAS research.

## Supporting Information

Additional supporting information can be found online in the Supporting Information section. **Supporting** **Fig.** **S1:** Calibration curve ammonium from 1 µg L^−1^ to 1000 µg L^−1^ (100 µL injection volume). **Supporting** **Fig.** **S2:** Sensitivity analysis of the calculated electrolyte ammonium concentration in µg L^−1^ as a function of the airborne ammonia contamination in ppb (temperature 22°C, electrolyte volume 32 mL). Both the contamination of 80 ppb_mol_ in the used nitrogen and the much lower local airborne ammonia contamination of 11 ppb_mol_ result in significant ammonium contamination liberated from the Nafion ionomer of the catalyst layer. **Supporting** **Fig.** **S3:** Sensitivity analysis of the calculated electrolyte ammonium concentration in µg L^−1^ as a function of percentage of the Henry's Law constant of ammonia at 22°C (ammonia contamination 80 ppb, electrolyte volume 32 mL). **Supporting** **Table** **S1:** Raw Data Calibration 1 µg L^−1^ – 100 µg L^−1^ Ammonium (100 µL injection). **Supporting** **Table** **S2:** Coefficients Analytical Calibration Function 1 µg L^−1^ – 100 µg L^−1^. **Supporting** **Table** **S3:** Raw Data Calibration 1 µg L^−1^ – 1000 µg L^−1^ Ammonium (100 µL injection). **Supporting** **Table** **S4:** Coefficients Analytical Calibration Function 1 µg L^−1^ – 1000 µg L^−1^. **Supporting** **Table** **S5:** Analysis of the chemical dissolution of ZrN catalyst powder in 0.2 M sulfuric acid electrolyte after 24 hours and quantitative determination of non‐catalytically produced ammonium by IC. **Supporting** **Table** **S6:** Ink composition for the manual spray‐coating of gas diffusion electrodes with synthesized zirconium nitride nanoparticles (target catalyst loading 1 mg·cm^−2^, 10 wt.‐% Nafion ionomer, catalyst concentration 36 mg mL^−1^). **Supporting** **Table** **S7:** Measurement protocol electrochemical turnover‐experiment using a ZrN GDE (0.2 M sulfuric acid electrolyte, gas flow 10 mL min^−1^). All voltages are versus the reversible hydrogen electrode (RHE). **Supporting** **Table** **S8:** Results the electrochemical dynamic turnover‐experiment of ZrN GDE (ZrN 0.95 mg cm^−2^, 10 wt.‐% Nafion ionomer, gas flow 10 mL min^−1^ nitrogen, room temperature). **Supporting** **Table** **S9:** Determined ammonia contamination in nitrogen 5.0 gas as is and purified by an Agilent in‐line gas purifier OT3‐4. **Supporting** **Table** **S10:** Annual average airborne ammonia pollution measured at the sampling stations ‘Südoldenburg’, ‘Allertal’ and ‘Emsland’. The data was taken from the air quality monitoring in lower saxony report 2023 table B10^[3]^.

## Conflicts of Interest

The authors declare no conflicts of interest.

## Supporting information

Supplementary Material
