# Supplementary material for: Development of Suppressed Ion Chromatography for the Online Quantification of Cations in Electrochemical Ammonia Synthesis Research
Source: ChemSusChem. 2026 Feb 2;19(3):e202501974. doi: 10.1002/cssc.202501974 (PMC12864167; doi:10.1002/cssc.202501974)
Supplement: Supplementary file 1 — Supplementary Material [file CSSC-19-e202501974-s001.pdf]

Supporting Information

Development of Suppressed Ion Chromatography for the Online Quantification of Cations in Electrochemical Ammonia Synthesis Research

Sebastian Bragulla*[a, c], Julian Lorenz[a], Corinna Harms[a], Michael Wark[d], K. Andreas Friedrich[b, c]

[a] S. C. H. Bragulla, Dr. J. Lorenz, Dr.-Ing. C. Harms
Electrochemical Energy Technology
Institute of Engineering Thermodynamics
Deutsches Zentrum für Luft- und Raumfahrt e. V. (DLR)
German Aerospace Center
Carl-von-Ossietzky-Straße 15, 26129 Oldenburg, Germany
E-Mail: Sebastian.Bragulla@dlr.de

[b] Prof. Dr. K. A. Friedrich
Electrochemical Energy Technology
Institute of Engineering Thermodynamics
Deutsches Zentrum für Luft- und Raumfahrt e. V. (DLR)
German Aerospace Center
Pfaffenwaldring 38-40, 70569 Stuttgart, Germany
E-Mail: Andreas.Friedrich@dlr.de

[c] S. C. H Bragulla, Prof. Dr. K. A. Friedrich
Institute for Building Energetics, Thermotechnology and Energy Storage (IGTE)
University of Stuttgart
Pfaffenwaldring 31, 70596 Stuttgart, Germany

[d] Prof. Dr. rer. nat. M. Wark
Faculty V – Mathematics and Natural Sciences
Institute of Chemistry

Chemical Technology 1

Carl von Ossietzky University

Carl-von-Ossietzky-Str. 9-11, 26129 Oldenburg, Germany

E-Mail: Michael.wark@uni-oldenburg.de

S1. Analytical Calibration Curve 1 µg·L^{-1} – 100 µg·L^{-1} Ammonium

Table S1 – Raw Data Calibration 1 µg·L^{-1} – 100 µg·L^{-1} Ammonium (100 µL injection)

Concentration c µg·L^{-1}	Amount Q ng	Peak Area A nS·cm^{-1}·min	ESD ΔA nS·cm^{-1}·min	Function Q_{calc} ng	Residual r^2 ng^2	Q_{xx} ng^2
1	0.1	0.412	0.063	0.053	0.002239	7.344
1	0.1	0.509	0.063	0.100	0.000000	7.344
1	0.1	0.531	0.063	0.110	0.000093	7.344
2	0.2	0.852	0.066	0.243	0.001831	6.812
2	0.2	0.839	0.066	0.238	0.001423	6.812
2	0.2	0.732	0.066	0.196	0.000020	6.812
4	0.4	1.411	0.062	0.437	0.001393	5.808
4	0.4	1.286	0.062	0.397	0.000012	5.808
4	0.4	1.353	0.062	0.419	0.000347	5.808
6	0.6	1.940	0.023	0.598	0.000003	4.884
6	0.6	1.894	0.023	0.585	0.000229	4.884
6	0.6	1.924	0.023	0.594	0.000039	4.884
8	0.8	2.719	0.076	0.812	0.000144	4.040
8	0.8	2.820	0.076	0.838	0.001478	4.040
8	0.8	2.867	0.076	0.851	0.002564	4.040
10	1	3.325	0.102	0.966	0.001159	3.276
10	1	3.437	0.102	0.993	0.000043	3.276
10	1	3.529	0.102	1.016	0.000251	3.276
20	2	7.450	0.493	1.870	0.016917	0.656
20	2	7.955	0.493	1.971	0.000866	0.656
20	2	8.435	0.493	2.065	0.004198	0.656
50	5	23.609	1.647	4.692	0.095116	4.796
50	5	25.529	1.647	4.997	0.000011	4.796
50	5	26.888	1.647	5.211	0.044333	4.796
80	8	44.326	1.175	7.837	0.026611	26.936
80	8	46.323	1.175	8.127	0.016205	26.936
80	8	46.399	1.175	8.138	0.019148	26.936
100	10	58.217	0.966	9.827	0.029841	51.696
100	10	59.654	0.966	10.030	0.000873	51.696
100	10	60.053	0.966	10.086	0.007347	51.696
**∑**					**0.274736**	**348.747**
**Ø**	**2.810**	**14.907**				

Calibration Function 1 µg·L^{-1} – 100 µg·L^{-1} Ammonium

$$x = f(y) = \beta_3 \cdot y + \beta_2 \cdot y^{\frac{1}{2}} + \beta_1$$

$$\frac{Q}{\mathrm{ng}} = 0.1078 \cdot \left(\frac{A}{\mathrm{nS \cdot cm^{-1} \cdot min}}\right) + 0.507 \cdot \left(\frac{A}{\mathrm{nS \cdot cm^{-1} \cdot min}}\right)^{\frac{1}{2}} - 0.317$$

Table S2 – Coefficients Analytical Calibration Function 1 µg·L^{-1} – 100 µg·L^{-1}

Coefficient	Value	Uncertainty	
β_3	0.1078	0.0044	4.1 %
β_2	0.507	0.022	4.3 %
β_1	-0.317	0.022	6.9 %

Calculation of the Method Limit of Detection 1 µg·L^{-1} – 100 µg·L^{-1}

$$x_{\mathrm{MLOD}} = \sqrt{\frac{RSS}{n-p}} \cdot t(\alpha, f) \cdot \sqrt{\frac{1}{m} + \frac{1}{n} + \frac{\bar{x}}{Q_{xx}}}$$

$$x_{\mathrm{MLOD}} = \sqrt{\frac{0.274736}{30-3}} \cdot t(0.95, 27) \cdot \sqrt{\frac{1}{1} + \frac{1}{30} + \frac{2.810^2}{348.747}}$$

$$x_{\mathrm{MLOD}} = \ 0.100873 \cdot 1.703288 \cdot 1.027606 \cong 0.177$$

$$c_{\mathrm{MLOD}} = 1.77\ \frac{\mathrm{\mu g}}{\mathrm{L}}, \qquad c_{\mathrm{MLOQ}} \cong c_{\mathrm{MLOD}} \cdot 3 \cong 5.3$$

S2. Analytical Calibration Curve 1 µg·L^{-1} – 1000 µg·L^{-1} Ammonium

Table S3 – Raw Data Calibration 1 µg·L^{-1} – 1000 µg·L^{-1} Ammonium (100 µL injection)

**Concentration**	**Amount**	**Peak Area**	**ESD**	**Function**	**Residual**	
**c**	**Q**	**A**	**ΔA**	**Q_{calc}**	**R^2**	**Q_{xx}**
**µg/L**	**ng**	**nS·cm^{-1}·min**	**nS·cm^{-1}·min**	**ng**	**ng^2**	**ng^2**
1	0.1	0.903	0.025	0.048	0.002740	373.842
1	0.1	0.854	0.025	0.032	0.004688	373.842
1	0.1	0.874	0.025	0.038	0.003830	373.842
2.5	0.25	1.527	0.039	0.233	0.000274	368.064
2.5	0.25	1.604	0.039	0.255	0.000021	368.064
2.5	0.25	1.554	0.039	0.241	0.000084	368.064
5	0.5	2.600	0.023	0.510	0.000104	358.534
5	0.5	2.641	0.023	0.520	0.000405	358.534
5	0.5	2.603	0.023	0.511	0.000118	358.534
10	1	5.008	0.078	1.049	0.002449	339.849
10	1	4.892	0.078	1.025	0.000623	339.849
10	1	4.860	0.078	1.018	0.000334	339.849
25	2.5	12.672	0.041	2.517	0.000301	286.794
25	2.5	12.753	0.041	2.532	0.001020	286.794
25	2.5	12.705	0.041	2.523	0.000549	286.794
50	5	27.367	0.187	5.014	0.000188	208.369
50	5	27.586	0.187	5.049	0.002442	208.369
50	5	27.213	0.187	4.989	0.000131	208.369
100	10	59.114	0.212	9.993	0.000053	89.019
100	10	58.742	0.212	9.936	0.004099	89.019
100	10	59.105	0.212	9.991	0.000074	89.019
250	25	159.832	2.351	24.878	0.014967	30.969
250	25	160.083	2.351	24.914	0.007374	30.969
250	25	164.025	2.351	25.487	0.236880	30.969
500	50	333.404	0.216	49.971	0.000845	934.219
500	50	333.819	0.216	50.031	0.000962	934.219
500	50	333.507	0.216	49.986	0.000201	934.219
1000	100	674.247	0.206	99.962	0.001432	6490.719
1000	100	674.656	0.206	100.023	0.000535	6490.719
1000	100	674.407	0.206	99.986	0.000193	6490.719
∑					**0.287915**	**28441.141**
**Ø**	**19.435**	**127.838**				

Calibration Function 1 µg·L^{-1} – 1000 µg·L^{-1} Ammonium

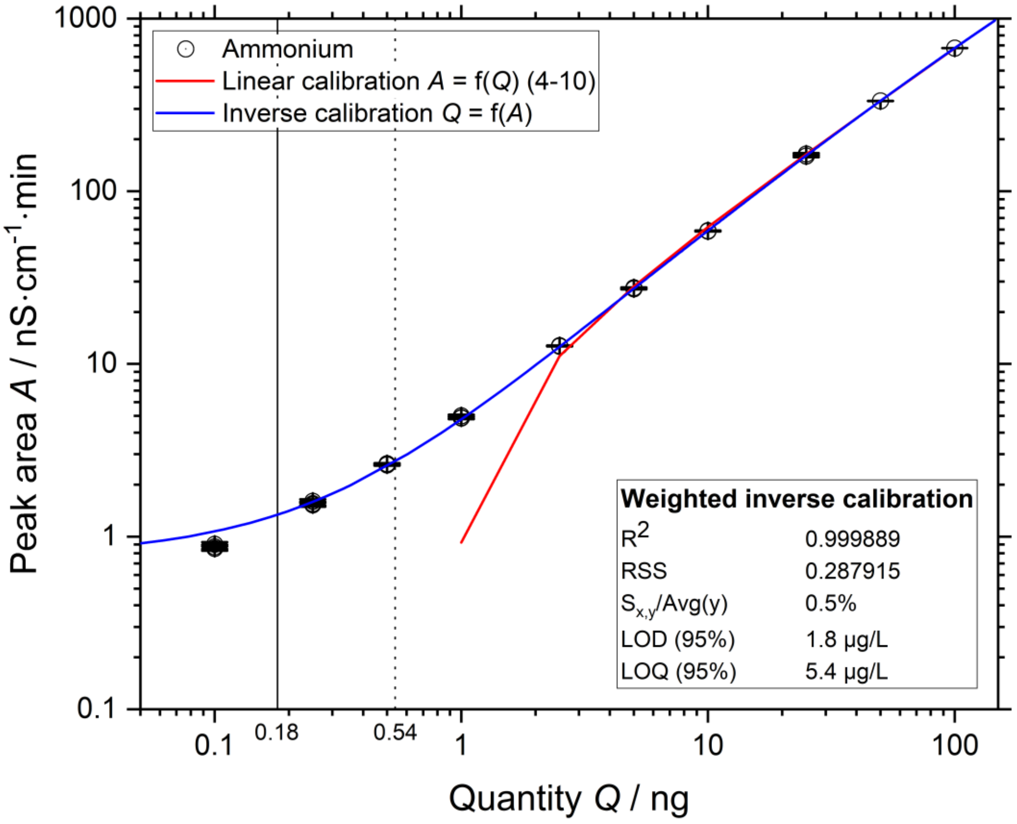

Figure S1 – Calibration curve ammonium from 1 µg·L^{-1} to 1000 µg·L^{-1} (100 µL injection volume)

$$x = f(y) = \beta_4 \cdot y^2 + \beta_3 \cdot y + \beta_2 \cdot y^{\frac{1}{2}} + \beta_1$$

$$\frac{Q}{\mathrm{ng}} = 1.115 \cdot 10^{-5} \cdot \left(\frac{A}{\mathrm{nS} \cdot \mathrm{cm}^{-1} \cdot \mathrm{min}}\right)^2 + 0.127 \cdot \left(\frac{A}{\mathrm{nS} \cdot \mathrm{cm}^{-1} \cdot \mathrm{min}}\right) + 0.373 \cdot \left(\frac{A}{\mathrm{nS} \cdot \mathrm{cm}^{-1} \cdot \mathrm{min}}\right)^{\frac{1}{2}} - 0.422$$

Table S4 – Coefficients Analytical Calibration Function 1 µg·L^{-1} – 1000 µg·L^{-1}

Coefficient	Value	Uncertainty	
β_4	$1.115 \cdot 10^{-5}$	$4.187 \cdot 10^{-7}$	3.8 %
β_3	0.127	$5.645 \cdot 10^{-4}$	0.4 %
β_2	0.373	$8.851 \cdot 10^{-3}$	2.4 %

β_1	-0.422	$2.783 \cdot 10^{-2}$	6.6 %

Calculation of the Method Limit of Detection 1 µg·L^{-1} – 1000 µg·L^{-1}

$$x_{\mathrm{MLOD}} = \sqrt{\frac{RSS}{n-p}} \cdot t(\alpha, f) \cdot \sqrt{\frac{1}{m} + \frac{1}{n} + \frac{\bar{x}}{Q_{xx}}}$$

$$x_{\mathrm{MLOD}} = \sqrt{\frac{0.287915}{30-4}} \cdot t(0.95, 26) \cdot \sqrt{\frac{1}{1} + \frac{1}{30} + \frac{19.435^2}{28441.141}}$$

$$x_{\mathrm{MLOD}} = 0.1052314 \cdot 1.705618 \cdot 1.023042 \cong 0.184$$

$$c_{\mathrm{MLOD}} = 1.84\ \frac{\mathrm{\mu g}}{\mathrm{L}}, \qquad c_{\mathrm{MLOQ}} \cong c_{\mathrm{MLOD}} \cdot 3 \cong 5.5$$

S3. Chemical Dissolution of ZrN Catalyst Powder

The chemical dissolution of nitrogen containing catalyst materials can result in the non-catalytic production of ammonium. The non-catalytic production of ammonium by chemical dissolution was investigated by aging pristine catalyst powder in 0.2 M sulfuric acid electrolyte for 24 hours and subsequent quantitative analysis of the electrolyte for ammonium. 15.1 mg catalyst powder were dispersed in 50 mL freshly prepared 0.2 M sulfuric acid electrolyte using a 50 mL volumetric flask made of PMP. The 50 mL volumetric flask was closed with a stopper and the electrolyte was stirred for 24 hours. An additional 50 mL volumetric flask was filled only with freshly prepared electrolyte and analyzed as baseline to compare against.

Table S5 – Analysis of the chemical dissolution of ZrN catalyst powder in 0.2 M sulfuric acid electrolyte after 24 hours and quantitative determination of non-catalytically produced ammonium by IC.

Mass	Volume	Concentration	Amount	Rate
mg	mL	$\mu g \cdot L^{-1}$ (n = 3)	µg	$pmol \cdot mg^{-1} \cdot s^{-1}$
15.1 ± 0.1 [a]	50 ± 0.12 [b]	45.1 ± 0.2 [c]	2.25 ± 0.03 [d]	0.08 ± 0.001 [d]

[a] Uncertainty of the used analytical scale e = 0.1 mg

[b] Uncertainty of the used 50 mL volumetric flask class B ± 0.12 mL

[c] Empirical standard deviation of the triplicate measurement

[d] Linear error propagation in mass, volume and concentration

The ammonium concentration in the electrolyte baseline was 1.2 $\mu g \cdot L^{-1}$, which is below the MLOD of 1.8 $\mu g \cdot L^{-1}$. The value of 1.2 $\mu g \cdot L^{-1}$ was subtracted from the result for further calculation nonetheless. The rate of non-catalytic ammonium production over the 24 hours is 0.08 ± 0.001 $pmol \cdot s^{-1}$ mg^{-1}, which is non-zero but negligible.

S4. Manual Spray-Coating of Gas Diffusion Electrodes

Gas diffusion electrodes (GDE) were spray-coated by hand using a professional airbrush pistol. The gas-diffusion-layer (GDL) material (Freudenberg H2315 C6 I2) with microporous layer (MPL) was cut into a square 8 cm x 8 cm. The cut-to-size GDL material was vertically fixed on a metal-plate with magnets using a thin metal-sheet stencil, leaving a central square 6 cm x 6 cm uncovered. The uncovered area was coated in a meandering line starting in one corner after another until all ink in the airbrush pistol was spent. The catalyst loading was controlled by the amount of catalyst ink decanted into the airbrush pistol. The amount of ink needed to reach the targeted catalyst loading of 1 mg·cm^{-2} was based on experience and was 3 to 4 times the theoretical value. The catalyst ink consisted of ZrN catalyst powder synthesized by carbothermal nitridation[1], Nafion ionomer, isopropanol and ultrapure water (see **Table S6**). The components were combined in order in a 15 mL centrifuge tube, briefly shaken by hand to combine and treated in an ultrasonic bath cooled with ice for 15 min. The ink was additionally treated with an ultrasonic horn equipped with a microtip for 15 mins using an ice bath (power 10 W·mL^{-1}, pulse duration 10 s, pause 10 s), after which the ink was immediately used. The deposited catalyst layer was tried in a desiccator. The gravimetric catalyst loading was 0.95 mg·cm^{-2}. The GDE was stored in another desiccator, which was constantly flushed with nitrogen. Individual Ø18 mm GDE samples were stamped out from the coated area.

Table S6 – Ink composition for the manual spray-coating of gas diffusion electrodes with synthesized zirconium nitride nanoparticles (target catalyst loading 1 mg·cm^{-2}, 10 wt.-% Nafion ionomer, catalyst concentration 36 mg·mL^{-1})

Component	Recipe		Actual
	mg	µL	mg
Catalyst powder	108		108
Ionomer D2021CS (1.02 g·mL^{-1})	57.1	56	59.6
Isopropanol (0.78 g·mL^{-1})	1122	1438	1128
Ultrapure water (0.997 g·mL^{-1})	1490	1494	1496.1

S5. Electrochemical Turnover-Experiment

The electrochemical turnover-experiments were conducted using a commercial Gaskatel FlexCell-PTFE measurement cell for GDEs in a three-electrode setup (active area Ø1.2 cm). The counter electrode (CE) was a spiral made of PtIr-wire supplied with the cell. A Gaskatel miniHydroflex reversible hydrogen electrode (RHE) was used as reference electrode. The electrochemical experiment was controlled by a Metrohm Autolab PGSTAT 128N potentiostat. The electrolyte consisted of 0.2 M sulfuric acid, freshly prepared by dilution of ultrapure 96 % sulfuric acid (Merck Supelco, Sulfuric acid, 96 %, ultrapure, 1.01516) with ultrapure water. The initial volume of electrolyte decanted into the cell was noted for later calculations. The used gases (nitrogen, argon) were cleaned by an Agilent in-line gas purifier OT3-4[2] and the gas flow of 10 mL·min^{-1} was controlled by a mass flow controller (MFC) (Sensirion, SFM5500-0.5slm). The assembled cell was flushed with argon for 15 min before start of electrochemical measurements. The electrolyte was constantly saturated with argon. The gas supplied to the GDE was switched to nitrogen at 10 mL·min^{-1} for the electrochemical measurements. Initial electrochemical characterization consisted of measuring the open-circuit potential (OCP) followed by electrochemical impedance spectroscopy (EIS). The catalyst was characterized by an initial cyclic voltammogram (CV), electrochemical cleaning by voltage sweeping and again a CV. The electrochemical activity of the catalyst was investigated by dynamically applying a reductive potential (-0.4, -0.6, -0.8 V vs. RHE) for 10 s, after which +0.6 V vs. RHE as resting potential was applied for 10 s. This was repeated for 2 hours total, starting with -0.4 V vs. RHE. The electrolyte was sampled before and after by drawing 2 mL of electrolyte for analysis by ion chromatography (IC).

Table S7 – Measurement protocol electrochemical turnover-experiment using a ZrN GDE (0.2 M sulfuric acid electrolyte, gas flow 10 mL·min^{-1}). All voltages are versus the reversible hydrogen electrode (RHE).

Operation	Name	Conditions	Purpose
1	OCP	Interval 0.1 s<br>Average 10 s<br>Duration 900 s	Determine OCP
2	EIS	0.1 Hz– 10 kHz<br>10 mV rms<br>10 points/dec	Measure iR-drop
3	CV	+0.6 – -0.6 V<br>100 mV·s^{-1}<br>3 scans	Initial characterization
4	EC cleaning	+0.6 – -0.6 V<br>250 mV·s^{-1}<br>50 scans	Remove surface oxides
5	CV	See 3	Check cleaning
6	Dynamic<br>-0.4 V –<br>+0.6 V	-0.4 V, 10 s<br>+0.6 V, 10 s<br>7200 s	Dynamic quantitative turnover at -0.4 V
7	Dynamic<br>-0.6 V –<br>+0.6 V	-0.6 V, 10 s<br>+0.6 V, 10 s<br>7200 s	Dynamic quantitative turnover at -0.6 V
8	Dynamic<br>-0.8 V –<br>+0.6 V	-0.8 V, 10 s<br>+0.6 V, 10 s<br>5400s	Dynamic quantitative turnover at -0.8 V

The electrochemical ammonia production rates at -0.4, -0.6 and -0.8 V vs. RHE were 1.34, 0.46 and 0.62 pmol·s^{-1}·mg^{-1} respectively (see Table S*8*), which is in the range of 1-3 pmol·s^{-1}·mg^{-1} we usually determined for nitrides in other experiments. The high ammonium concentration after initial electrochemical characterization was ascribed to the reduction of nitrate contamination during the initial CV. However, the liberation of

ammonium contamination from the Nafion ionomer of the catalyst layer is an equally suitable explanation.

Table S8 – Results the electrochemical dynamic turnover-experiment of ZrN GDE (ZrN 0.95 mg·cm^{-2}, 10 wt.-% Nafion ionomer, gas flow 10 mL·min^{-1} nitrogen, room temperature)

			Electrolyte	Initial EC	activated EC	-0.4 V	-0.6 V	-0.8 V
Concentration	c	µg·L^{-1}	<0.5	47.4	60.7	71.6	79.3	88.8
Volume	V	mL	30	30	28	26	24	22
Corrected concentration	c'	µg·L^{-1}			59.8	70.8	78.7	88
Molar quantity	n	nmol	0.83	78.1	99.5	109.9	113.5	117.1
Difference	Δn	nmol		78.1	21.41	10.40	3.57	3.60
Step duration	Δt	s		1272	562	7200	7200	5400
Rate	r	pmol·s^{-1}·cm^{-2}		54.27	33.69	1.28	0.44	0.59
		pmol·s^{-1}·mg^{-1}		57.13	35.46	1.34	0.46	0.62

S6. Contamination of Nafion Dispersion Ion Power D2021CS

The Nafion dispersion Nafion™ D2021CS (Ion Power, alcohol based, 20 wt.-% Nafion, 1100 EW) was used for preparation of hand-sprayed gas-diffusion electrodes (GDE). The Nafion dispersion was used as delivered and stored in the original container wrapped with Parafilm. A set amount of dispersion was pipetted into an IC sample tube and liquid was added to achieve a concentration of 19.1 mg_{D2021}/mL with a total solvent volume of 3 mL. While the ink used for hand-spraying consists of a 1:1 volume-mixture of isopropanol and ultrapure water, two additional samples were prepared with 0.2 M sulfuric acid and ultrapure water as matrix. 0.2 M sulfuric acid was chosen because it is used as electrolyte, and the high proton concentration should liberate all retained cationic contamination. There was no significant ammonium content in any of the three samples. Investigating Nafion dispersion by IC was problematic due to permanent contamination of the system with Nafion, which interferes with separation and quantification of any subsequent measurement.

S7. Ammonia Contamination in used Nitrogen 5.0 Gas Supply

The ammonia contamination of the used nitrogen 5.0 house gas supply was investigated using two gas-washing bottles in series to dissolve any present ammonia. Two micro-gas-washing bottles made of PFA (manufacturer BOLA) with a volume of 50 mL were each cleaned, filled with freshly prepared 0.2 M sulfuric acid and connected in series with 6 mm PTFE tubing. The gas flow was set to 10 $mL{\cdot}min^{-1}$ and was controlled by a thermal mass flow controller (MFC) (Sensirion, SFM5500-0.5slm). The measured gas flow was recorded and integrated to calculate the total gas volume that passed through the gas-washing bottles. The gas flow was set to 10 $mL{\cdot}min^{-1}$ because it is used in EC turnover experiments. A gas flow of 10 $mL{\cdot}min^{-1}$ is sufficiently low for full retention of gaseous contamination by the gas-washing bottles[2]. The experimental conditions are given in **Table S9**.

Table S9 – Determined ammonia contamination in nitrogen 5.0 gas as is and purified by an Agilent in-line gas purifier OT3-4.

	Duration h	Volume L	Concentration $\mu g{\cdot}L^{-1}$	Amount µg	Contamination ppb_{mol}	Rate $pmol{\cdot}s^{-1}$
N_2 5.0	21.53	12.92	14.8 / 0.4	0.763	78.8 ± 5.1	0.55
N_2 5.0, purified	64.32	38.57	1.3 / 0.4	0.084	2.9 ± 2.2	0.02

The experimental duration was increased for the purified gas because the expected ammonium concentration was much lower. Although the duration was increased to more than 60 hours, the measured ammonium concentrations were still below the MLOD of 1.8 $\mu g{\cdot}L^{-1}$. Nevertheless, the concentrations were evaluated to give an estimate. The ammonia contamination of the used nitrogen 5.0 house supply was rounded up to 80 ppb_{mol}. The Agilent OT3-4 gas purifier reduced this ammonia contamination to approximately 3 ppb_{mol}, a reduction of 93-99 %. The stated uncertainty was calculated by a linear error propagation of uncertainties in the measured concentration, liquid volume, flow rate and duration.

S8. Equilibrium Ammonia Contamination Uptake of the GDE

Local Annual-Average Ammonia Air Pollution

The used laboratories are located in Oldenburg, Lower Saxony, Germany. The States Inspectorate's Office Hildesheim publishes an annual report on the air quality monitoring in Lower Saxony (German 'Luftqualitätsüberwachung in Niedersachsen'). The following data has been taken from the 2023 report, which can be downloaded at https://www.umwelt.niedersachsen.de/download/208805/Jahresbericht_2023.pdf (15.07.2025, 07:57). The sampling station 'Südoldenburg' is located the closest to the urban area of Oldenburg. This station is designated as in the vicinity of industry (German 'Industriestation'). The sampling stations 'Allertal' and 'Emsland' are designated as background stations and are located close to the urban area of Oldenburg and the sampling station 'Südoldenburg' respectively. The annual average airborne ammonia pollution for these sampling stations is given in Table S10[3].

Table S10 – Annual average airborne ammonia pollution measured at the sampling stations 'Südoldenburg', 'Allertal' and 'Emsland'. The data was taken from the air quality monitoring in lower saxony report 2023 table B10[3].

Name	Classification	Annual average airborne ammonia	
		$\mu g \cdot m^{-3}$	ppb_{mol} [a]
Südoldenburg	Suburban, industry	7.7	10.9
Allertal	Suburban, background	2.8	4
Emsland	Suburban, background	3.6	5.1

[a] Calculated by ideal gas law using a temperature of 22 °C and a pressure of 1 atm.

Conversion of airborne ammonia concentration from $\mu g \cdot m^{-3}$ to ppb_{mol} was done via the following equation:

$$\frac{\mu g_{NH_3}}{m_{Air}^3} = \frac{\frac{\mu g_{NH_3}}{M_{NH_3}}}{\frac{m_{Air}{}^3}{V_M}} = \frac{\mu g_{NH_3}}{m_{Air}^3} \cdot \frac{V_M}{M_{NH_3}}$$

$$V_M = \frac{V}{n} = \frac{R \cdot T}{p} = \frac{8.314462 \cdot (273.15 + 22)}{1.01325 \cdot 10^5} \; \frac{\frac{Nm}{mol \cdot K} \cdot K}{\frac{N}{m^2}} = 0.0242 \; \frac{m^3}{mol}$$

$$7.7 \; \frac{\mu g}{m^3} \cdot \frac{0.0242}{17.0307} \frac{\frac{m^3}{mol}}{\frac{g}{mol}} = 10.94 \text{ ppb}$$

Calculation of the Equilibrium Ammonium Uptake

We assume that the Nafion inside the catalyst layer of the hand-sprayed GDE retains some water after spray-coating and drying in a desiccator, which serves as first reservoir for the ingress of gaseous ammonia from present environmental contamination. Therfore, we assume that the transport processes can be described using Henry's law, ideal gas law, and are in equilibrium. The equilibrium ammonium content of Nafion is calculated based on the data by Hongsirikarn et al[4]. The Henry's Law data was taken from Burkolder et al.[5]. The laboratories are climate controlled, an average temperature of 22 °C and an atmospheric pressure of 1 atm is used for calculations.

First, we calculate the pH of retained ultrapure water after equilibration with the CO_2 in the atmosphere. The monthly average Mauna Loa atmospheric CO_2 in June 2024 was 427 ppm as published by the NOAA Global Monitoring Laboratory[6]. The Henry's Law constant for CO_2 in pure water in the temperature range of 273 to 353 K is given as

$$\frac{H_{CO_2}}{\frac{M}{atm}} = \exp\left(-123.3 + \frac{7335}{T} + 16.739 \cdot \ln(T)\right)$$

with an uncertainty class I of better than 10 %[5]. The Henry's Law constant for CO_2 in pure water at 22 °C is 0.038951 $M \cdot atm^{-1}$.

$$\frac{H_{CO_2}}{\frac{\mathrm{M}}{\mathrm{atm}}} = \exp\left(-123.3 + \frac{7335}{295.15} + 16.739 \cdot \ln(295.15)\right) \cong 0.038951$$

The concentration of dissolved CO_2 is

$$c_{\mathrm{CO_2,aq}} = p \cdot x_{\mathrm{CO_2}} \cdot H_{\mathrm{CO_2}} = 1\ \mathrm{atm} \cdot 427\ \mathrm{ppm} \cdot 0.038951\ \frac{\mathrm{M}}{\mathrm{atm}} = 16.63\ \mu\mathrm{M}$$

We calculate the pH of the ultrapure water based on the concentration of dissolved CO_2. The proton concentration is calculated using the pK_a of 6.35[7].

$$\mathrm{K_{a,CO_2}} = 10^{-\mathrm{pK_{s,CO_2}}} = 10^{-6.35} \cong 4.467 \cdot 10^{-7}\ \frac{\mathrm{mol}}{\mathrm{L}}$$

$$\mathrm{K_{a,CO_2}} = \frac{[\mathrm{H_3O^+}] \cdot [\mathrm{HCO_3^-}]}{[\mathrm{CO_2}]} \Leftrightarrow [\mathrm{H_3O^+}] = \sqrt{\mathrm{K_{a,CO_2}} \cdot c_{\mathrm{CO_2}}}$$

$$[\mathrm{H_3O^+}] = \sqrt{10^{-6.35} \cdot 16.63 \cdot 10^{-6}} \cdot \frac{\mathrm{mol}}{\mathrm{L}} \cong 2.73 \cdot 10^{-6}\ \frac{\mathrm{mol}}{\mathrm{L}}$$

$$\mathrm{pH} = -\log_{10}\left(\mathrm{a(H_3O^+)}\right) \approx -\log_{10}([\mathrm{H_3O^+}]) = -\log_{10}\left(2.73 \cdot 10^{-6} \cdot \frac{\mathrm{mol}}{\mathrm{L}}\right) \cong 5.56$$

The calculated pH of 5.56 is consistent with reported values for ultrapure water. The uncertainty of the used Henry's Constant is less than 10 %. Varying the calculated concentration by 10 % in either direction gives a pH of 5.54 and 5.59.

The Henry's Law constant for NH_3 in pure water in the temperature range of 273 to 348 K is given as

$$\frac{H_{\mathrm{NH_3}}}{\frac{\mathrm{M}}{\mathrm{atm}}}(295.15) = \exp\left(-9.84 + \frac{4160}{295.15}\right) = 70.4$$

with an uncertainty class III of 50 % to 100 %[5]. While the solubility of ammonia in water is much more complex in the presence of dissolved CO_2[8], the simplified calculation is an estimate of the environmental ammonia contamination liberated from the Nafion ionomer. Based on the determined ammonia contamination of approximately 80 ppb in the used nitrogen, the amount of dissolved ammonia in ultrapure water is

$$c_{\mathrm{NH3}} = 1\,\mathrm{atm} \cdot 80\,\mathrm{ppb} \cdot 70.4\,\frac{\mathrm{M}}{\mathrm{atm}} = 5.6 \cdot 10^{-6} \cdot \frac{\mathrm{mol_{NH_3}}}{\mathrm{L}} \rightarrow 95.2\,\frac{\mathrm{\mu g_{NH_3}}}{\mathrm{L}}.$$

The fraction of ammonium is calculated based on the ammonia pK_b of 4.75[7].

$$\mathrm{NH_3 + H_2O \overset{K_{b,NH_3}}{\Longleftrightarrow} NH_4^+ + OH^-}$$

$$\mathrm{K_{b,NH_3}} = 10^{-4.75} \cong 1.78 \cdot 10^{-5}$$

$$\mathrm{K_{b,NH_3}} = \frac{[\mathrm{NH_4^+}] \cdot [\mathrm{OH^-}]}{[\mathrm{NH_3}]} \Leftrightarrow [\mathrm{NH_4^+}] = \sqrt{\mathrm{K_{b,NH_3}} \cdot [\mathrm{NH_3}]}$$

$$c_{\mathrm{NH_4^+,aq}} = \sqrt{10^{-4.75} \cdot 5.6 \cdot 10^{-6} \cdot \frac{\mathrm{mol}}{\mathrm{L}}} \cong 10\,\frac{\mathrm{\mu mol}}{\mathrm{L}}$$

$$x = \frac{[\mathrm{NH_4^+}]}{[\mathrm{NH_4^+}] + [\mathrm{H_3O^+}]} = \frac{10 \cdot 10^{-6}}{10 \cdot 10^{-6} + 2.73 \cdot 10^{-6}} \cong 0.786$$

The data on the equilibrium ammonium content of Nafion has been taken from Hongsirikarn et al.[4]. The digitalized data was fitted using a 4. Order polynomial:

$$y = -1.8014 \cdot x^4 + 4.5128 \cdot x^3 - 4.3261 \cdot x^2 + 2.6236 \cdot x - 0.0049$$

$$y(x = 0.786) \cong 0.888 = \frac{n_{\mathrm{NH_4^+}}}{\mathrm{IEC}}$$

$$n_{\mathrm{NH_4^+}} = \mathrm{IEC} \cdot y \cdot \left(1 - \frac{\mathrm{n_{Ca^{2+}}}}{\mathrm{IEC}}\right) = 0.255 \cdot 0.909 \cdot (1 - 0.196) \cdot \mathrm{\mu mol} = 0.182\,\mathrm{\mu mol}$$

$$c_{NH_4^+,El} = \frac{\left(0.182\ \mu mol \cdot 18.0387\ \frac{g}{mol}\right)}{32 \cdot 10^{-3}\ L} \cong 103\ \frac{\mu g}{L}$$

Varying the Henry's Law constant by ± 50 % yields an ammonium concentration of 97 µg·L^{-1} and 105 µg·L^{-1} assuming total liberation from the Nafion ionomer. A sensitivity analysis was carried out using the following equations. The results for varying the airborne ammonia contamination and the Henry's Law constant are shown in Figure S2 and Figure S3 respectively.

$$c_{NH_4^+,El} = f(y) = (EC \cdot y) \cdot \frac{M}{V}$$

$$y = f(x) = -1.8014 \cdot x^4 + 4.5128 \cdot x^3 - 4.3261 \cdot x^2 + 2.6236 \cdot x - 0.0049$$

$$x = f(\chi) = \frac{c_{NH_4^+,aq}}{c_{NH_4^+,aq} + c_{H^+,aq}} = \frac{1}{1 + \frac{c_{H^+,aq}}{c_{NH_4^+,aq}}} = \left(1 + \frac{c_{H^+,aq}}{c_{NH_4^+,aq}}\right)^{-1} = (1+\chi)^{-1}$$

$$\chi = f(x_{CO_2}, x_{NH_3}, T) = \frac{c_{H^+,aq}}{c_{NH_4^+,aq}} = \frac{\left(p \cdot x_{CO_2} \cdot H_{CO_2} \cdot k_{A,CO_2}\right)^{\frac{1}{2}}}{\left(p \cdot x_{NH_3} \cdot H_{NH_3} \cdot k_{B,NH_3}\right)^{\frac{1}{2}}}$$

$$\chi = \frac{\left(x_{CO_2} \cdot \exp\left(-123.3 + \frac{7335}{T} + 16.739 \cdot \ln(T)\right) \cdot 10^{-6.25}\right)^{\frac{1}{2}}}{\left(x_{NH_3} \cdot \exp\left(-9.84 + \frac{4160}{T}\right) \cdot 10^{-4.75}\right)^{\frac{1}{2}}}$$

$$\chi = \left(\frac{x_{CO_2}}{x_{NH_3}}\right)^{\frac{1}{2}} \cdot 10^{-0.75} \cdot \left(\frac{\exp\left(-123.3 + \frac{7335}{T} + 16.739 \cdot \ln(T)\right)}{\exp\left(-9.84 + \frac{4160}{T}\right)}\right)^{\frac{1}{2}}$$

$$\chi = \left(\frac{\frac{427}{80}}{1000}\right)^{\frac{1}{2}} \cdot 10^{-0.75} \cdot \left(\frac{\exp\left(-123.3 + \frac{7335}{295.15} + 16.739 \cdot \ln(295.15)\right)}{\exp\left(-9.84 + \frac{4160}{295.15}\right)}\right)^{\frac{1}{2}}$$

$$\chi = 73.06 \cdot 0.17783 \cdot 0.02352 = 0.305578$$

$$x = (1+\chi)^{-1} = (1 + 0.305578)^{-1} = 0.7659$$

$$y = -1.8014 \cdot 0.7659^4 + 4.5128 \cdot 0.7659^3 - 4.3261 \cdot 0.7659^2 + 2.6236 \cdot 0.7659 - 0.0049$$

$$y = -0.62 + 2.03 - 2.54 + 2.01 - 0.0049 = 0.8751$$

$$n_{\mathrm{NH_4^+}} = 0.255 \cdot 0.8751 \cdot (1 - 0.196)\ \mu\mathrm{mol} = 0.179\ \mu\mathrm{mol}$$

$$c_{\mathrm{NH_4^+,El}} = \frac{\left(0.179\ \mu\mathrm{mol} \cdot 18.0387\ \frac{\mathrm{g}}{\mathrm{mol}}\right)}{32 \cdot 10^{-3}\ \mathrm{L}} \cong 101\ \frac{\mu\mathrm{g}}{\mathrm{L}}$$

Sensitivity Analysis

Figure S2 shows the sensitivity analysis of the calculated ammonium electrolyte concentration as a function of the airborne ammonia contamination. The asymptotic line clearly shows that minute airborne ammonia contamination will results in significant ammonium concentrations liberated from the Nafion ionomer in the catalyst layer. Even a contamination of 10 ppb in the air, as determined for the geographical region in 2023, would result in an ammonium electrolyte concentration of 84 µg·L^{-1}. This illustrates that Nafion containing GDEs will have to be cleaned before use, if the catalyst layer contains Nafion.

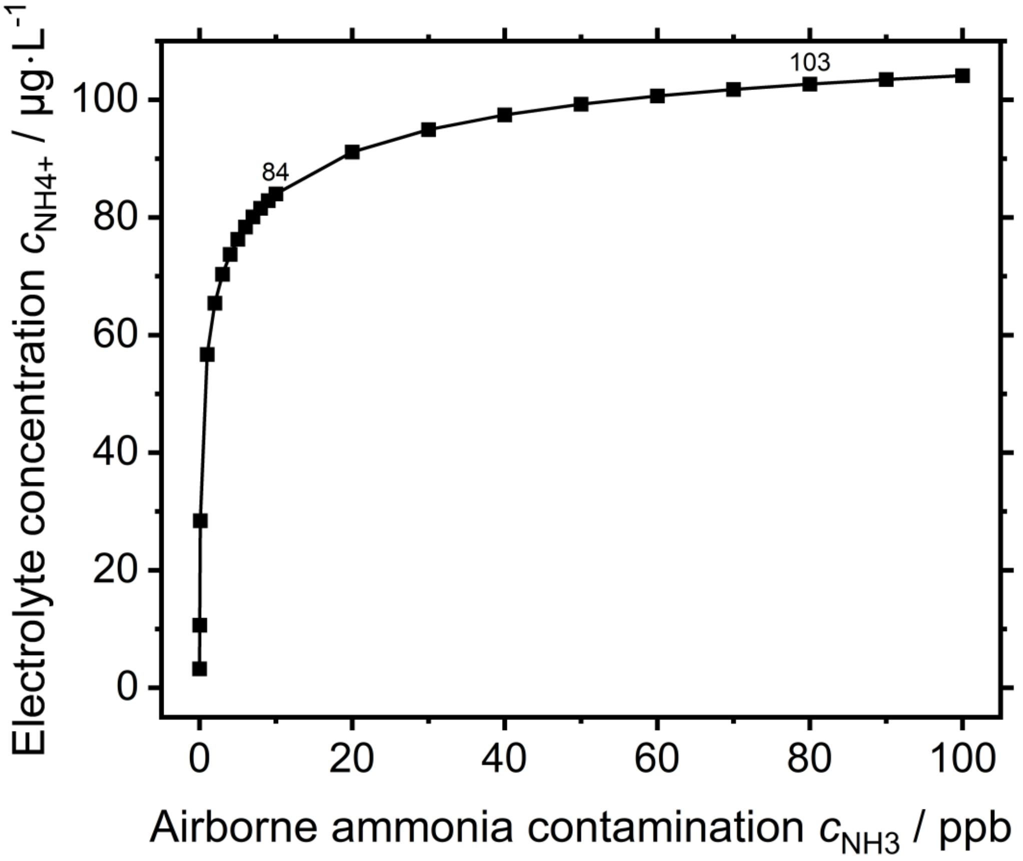

Figure S2 – Sensitivity analysis of the calculated electrolyte ammonium concentration in µg·L^{-1} as a function of the airborne ammonia contamination in ppb (temperature 22 °C, electrolyte volume 32 mL). Both the contamination of 80 ppb$_{mol}$ in the used nitrogen and the much lower local airborne ammonia contamination of

11 ppb_{mol} result in significant ammonium contamination liberated from the Nafion ionomer of the catalyst layer.

The effect of varying the Henry's Law constant for ammonia on the calculated electrolyte ammonium concentration is shown in Figure S3. It illustrates that although the used Henry's Law data has an uncertainty range of 50-100 %[5], the impact on the calculated electrolyte ammonium is low at an airborne ammonia contamination of 80 ppb, with the exception of a diminishingly low Henry's Law constant, where the value is reduced to 54 $\mu g \cdot L^{-1}$. However, even this concentration is significant for false-positive results in eNRR research.

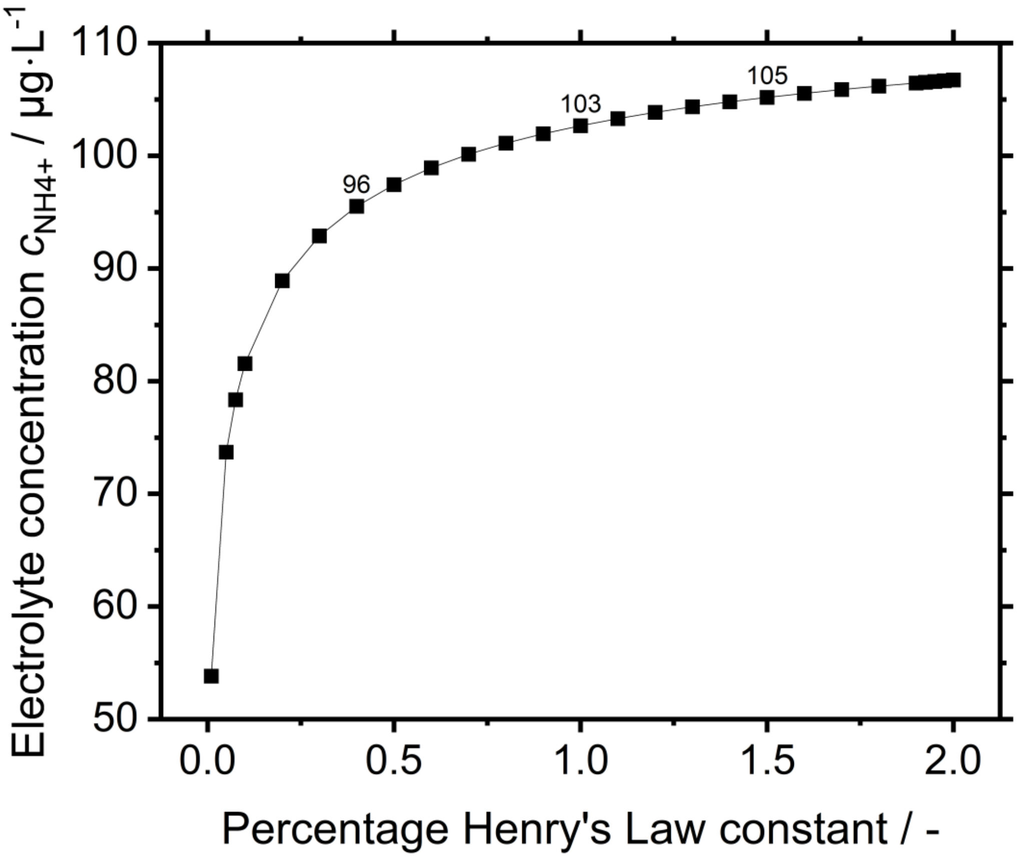

Figure S3 – Sensitivity analysis of the calculated electrolyte ammonium concentration in $\mu g \cdot L^{-1}$ as a function of percentage of the Henry's Law constant of ammonia at 22 °C (ammonia contamination 80 ppb, electrolyte volume 32 mL).

[1] S. C. H. Bragulla, A. R. von Seggern, J. Lorenz, C. Harms, M. Wark, K. A. Friedrich, *ChemCatChem* **2024**, *16*.

[2] B. Izelaar, D. Ripepi, D. D. van Noordenne, P. Jungbacker, R. Kortlever, F. M. Mulder, *ACS energy letters* **2023**, *8*, 3614.

[3] *Luftqualitätsüberwachung in Niedersachsen*. *Jahresbericht 2023*, Hildesheim, **2024**.

[4] K. Hongsirikarn, J. G. Goodwin, S. Greenway, S. Creager, *Journal of Power Sources* **2010**, *195*, 30.

[5] S. B. Burkholder, S. P. Sander, R. Barker, C. Cappa, J. D. Crounse, T. S. Dibble, R. E. Huie, C. E. Kolb, M. J. Kurylo, Orkin, V. L., Percival, J. C. et al., *Chemical kinetics and photochemical data for use in atmospheric studies; evaluation number 19*, **2020**.

[6] NOAA Global Monitoring Laboratory, "Trends in Atmospheric Carbon Dioxide (CO2). Weekly average CO2 at Mauna Loa", can be found under https://gml.noaa.gov/ccgg/trends/weekly.html, **2025**.

[7] E. Riedel, *Anorganische Chemie*. *6. Auflage*, de Gruyter, Berlin, **2004**.

[8] J. M. Hales, D. R. Drewes, *Atmospheric Environment (1967)* **1979**, *13*, 1133.
